# TCF21^+^ mesenchymal cells contribute to testis somatic cell development, homeostasis, and regeneration in mice

**DOI:** 10.1038/s41467-021-24130-8

**Published:** 2021-06-23

**Authors:** Yu-chi Shen, Adrienne Niederriter Shami, Lindsay Moritz, Hailey Larose, Gabriel L. Manske, Qianyi Ma, Xianing Zheng, Meena Sukhwani, Michael Czerwinski, Caleb Sultan, Haolin Chen, Stephen J. Gurczynski, Jason R. Spence, Kyle E. Orwig, Michelle Tallquist, Jun Z. Li, Saher Sue Hammoud

**Affiliations:** 1 Department of Human Genetics, University of Michigan, Ann Arbor, MI USA; 2 Cellular and Molecular Biology Program, University of Michigan, Ann Arbor, MI USA; 3 Department of Obstetrics, Gynecology and Reproductive Sciences, Integrative Systems Biology Graduate Program, Magee-Womens Research Institute, University of Pittsburgh School of Medicine, Pittsburgh, PA USA; 4 Department of Internal Medicine, University of Michigan, Ann Arbor, MI USA; 5 Biochemistry and Molecular Biology, Bloomberg School of Public Health, John Hopkins, USA; 6 University of Hawaii, Center for Cardiovascular Research, Honolulu, HI USA; 7 Department of Computational Medicine and Bioinformatics, University of Michigan, Ann Arbor, MI USA; 8 Department of Obstetrics and Gynecology, University of Michigan, Ann Arbor, MI USA; 9 Department of Urology, University of Michigan, Ann Arbor, MI USA

**Keywords:** Germline development, RNA sequencing, Transcriptomics, Reproductive biology, Mesenchymal stem cells

## Abstract

Testicular development and function rely on interactions between somatic cells and the germline, but similar to other organs, regenerative capacity declines in aging and disease. Whether the adult testis maintains a reserve progenitor population remains uncertain. Here, we characterize a recently identified mouse testis interstitial population expressing the transcription factor Tcf21. We found that TCF21^lin^ cells are bipotential somatic progenitors present in fetal testis and ovary, maintain adult testis homeostasis during aging, and act as potential reserve somatic progenitors following injury. In vitro, TCF21^lin^ cells are multipotent mesenchymal progenitors which form multiple somatic lineages including Leydig and myoid cells. Additionally, TCF21^+^ cells resemble resident fibroblast populations reported in other organs having roles in tissue homeostasis, fibrosis, and regeneration. Our findings reveal that the testis, like other organs, maintains multipotent mesenchymal progenitors that can be potentially leveraged in development of future therapies for hypoandrogenism and/or infertility.

## Abstract (web summary)

Whether the adult testis harbours a somatic progenitor population is unknown. Here, the authors provide evidence that the testis interstitial cells expressing the transcription factor Tcf21 maintain adult testis homeostasis during aging, and act as potential reserve somatic progenitors following injury.

## Introduction

Sexual reproduction relies on the generation of distinct sexes to increase biological diversity. The core of this strategy rests with a bipotential gonadal primordium that supports development of sex-specific reproductive organs with functionally distinct gonadal cell types. The gonadal primordium is comprised of primordial germ cells and mesenchymal cells that originate from two sources: the coelomic epithelium^1–5^ and the mesonephros^6^. In early development, the coelomic epithelial cells give rise to multiple cell lineages, including interstitial cells and Sertoli cells^1^, but later become restricted to the interstitial compartment of the testis. In contrast, mesonephric-derived cells migrating into the gonad contribute only to fetal/adult Leydig and interstitial cells, but not Sertoli cells^7,8^. Hence, the gonadal mesenchyme is heterogeneous on the molecular and cellular scales, and certain somatic lineages (e.g. Leydig and myoid) have multiple cells of origin^9^, pointing to a complex developmental programming of reproductive organs (reviewed in^10–12^).

Establishment of a functional somatic microenvironment is essential for continuous sperm production, germ cell homeostasis, and regeneration^13–15^. Disruptions in somatic cell populations can dramatically alter germ cell development and testis function. For instance, genetic ablation of macrophages in the adult testis leads to disruption of spermatogonial proliferation and differentiation^16^. Others have shown that testicular endothelial cells^14^ as well as lymphatic endothelial cells^15^ support human and mouse spermatogonial stem cell survival and expansion, and can also modulate spermatogonial cell homeostasis and regeneration^15^. Therefore, these studies underscore the importance of defining the interstitial cell composition and function.

The testis interstitial cells are believed to be postmitotic (not actively proliferating). However, studies in rats have demonstrated that adult Leydig cells can regenerate after ethane dimethane sulfonate (EDS)-induced cell death (reviewed in^17^). Multiple interstitial cell (CD90/PDGFRA/COUPTFII/NESTIN) populations were shown to re-enter the cell cycle upon EDS-induced Leydig cell death, suggesting that the interstitial compartment contains a reserve Leydig or a general somatic cell progenitor population that can be activated in response to damage^18–26^. Without single-cell RNA-seq analysis it remains difficult to tease apart whether these markers are observed in a single homogenous population of Leydig stem cells or if these cells are a heterogenous pool of progenitors. Furthermore, it is also unclear if a common somatic progenitor could give rise to additional cell types other than Leydig, and what the role of such stem/progenitors would be in the normal adult testis.

Using single-cell RNA sequencing (scRNA-seq) we previously identified a mesenchymal cell population in the adult mouse testis that expresses the transcription factor *Tcf21* and appears in vivo as rare spindle-shaped cells surrounding the seminiferous tubule^13^. The *Tcf21* gene has known roles in the development of multiple organs, including the testis^27–29^. Loss of Tcf21 *Tcf21* promotes feminization of external genitalia in karyotypically male mice^30^, while overexpression of Tcf21 in primary embryonic ovary cells leads to in vitro sex-reversal via aberrant anti-Mullerian hormone expression^31^. These results provide evidence for a role of TCF21 in male sex determination and testis somatic cell differentiation. Recent reports of single-cell sequencing during sex determination and cell linage specification also identified *Tcf21* expression among subsets of gonadal somatic cells in both male and female, although these experiments were limited to NR5A1-eGFPcells^32,33^. The similarity between the adult *Tcf21*^+^ population and fetal somatic progenitors^32,33^ suggests that the *Tcf21*^+^ population that persists in adulthood may retain fetal developmental or functional properties.

In this study, we examine the role of *TCF21*^+^ cells in the developing and adult mouse testis. To answer this question, we utilize genetic lineage tracing to mark and follow the potential and fate of the TCF21^lin^ population both in vitro and in vivo. First, in directed in vitro differentiation paradigms, we find that flow-sorted TCF21^lin^ cells possess mesenchymal stem cell (MSC)-like properties and can be directed to differentiate to either myoid or Leydig cell lineages, thus acting as true multipotent progenitors in vitro. In vivo, fetal TCF21^lin^ cells are bipotential somatic progenitors, contributing to all known somatic cell populations in the fetal and adult testis and ovary. In the adult testis, the TCF21^lin^ cells replenish somatic populations in response to injury as well as in normal aging. Furthermore, the adult testis *Tcf21*^+^ cells resemble resident fibroblast populations in multiple organs which have been implicated in tissue homeostasis, fibrosis, and regeneration. In summary, our work demonstrates the first evidence for a reserve somatic cell population in the adult testis, representing a potential targetable cell population for development of treatments for gonad-related defects and disease.

## Results

### The *Tcf21*^+^ population is a molecularly heterogenous mesenchymal cell population that is transcriptomically similar to myoid and Leydig cells

**Fig. 1:**
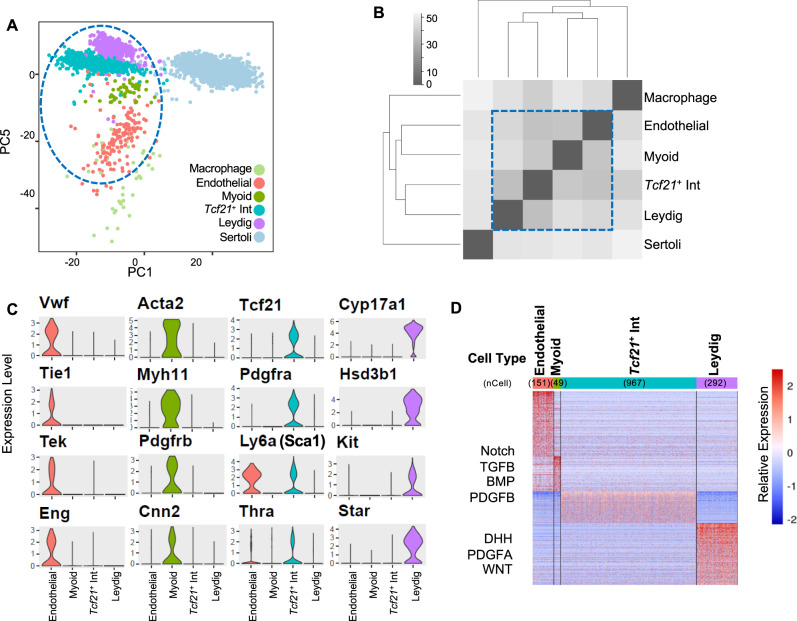
Identification and characterization of a *Tcf21*-expressing interstitial somatic cell in the adult testis. **A** Visualization of 6 somatic cell types in PC space. Data reprocessed from Green et al.^13^. Note somatic cell populations were enriched using a combination of cell surface markers or transgenic lines, therefore cell frequencies in PCA plot are not representative of in vivo. **B** Heatmap of dissimilarity matrix of somatic cell types illustrates high similarity for the *Tcf21*^*+*^ interstitial population with 3 somatic cell types—myoid, Leydig, and endothelial cells. Gray scale indicates Euclidean distance. **C** Violin plots of representative markers for endothelial, myoid, *Tcf21*^*+*^ interstitial population, and Leydig cells. **D** Heatmap of differentially expressed markers for each of the 4 closely related somatic cell types—endothelial, myoid, *Tcf21*^*+*^ interstitial population, and Leydig cells.

We recently employed single-cell RNA-seq (scRNA-seq) to generate a cell atlas of the mouse testis^13,34^. Our analysis of ~35,000 cells identified all known somatic cell types as well as an unexpected *Tcf21*^+^ population (Fig. 1A)^13^. To better understand its potential function, we first examined if the *Tcf21*^+^ population has molecular similarity to other known somatic cells in the testis. By using a pairwise dissimilarity matrix for somatic cell centroids, we find that the *Tcf21*^+^ population was distinct from macrophage and Sertoli lineages but transcriptomically similar to myoid, Leydig, and endothelial cells (Fig. 1B). In contrast with these cell types, *Tcf21*^+^ cells did not express any of their terminally differentiated markers (Fig. 1C). Rather, this population was uniquely demarcated by the expression of *Tcf21*, *Pdgfra*, *CoupTFII*, and mesenchymal progenitor cell (MP) markers including *Sca1* (Fig. 1C, Supplementary Data 1), *Arx*, and *Vim*^13^. To define molecular properties of *Tcf21*^+^ cells more broadly we identified differentially expressed genes between the *Tcf21*^+^ population and its most similar cell types (using >2-fold change and FDR < 5%). Gene ontology analysis suggested that the *Tcf21*^+^ population is of mesenchymal origin, and likely involved in extracellular matrix (ECM) biology, tissue injury, and repair processes (Fig. 1D; Supplementary Data 1). Although the ECM was once considered a passive support scaffold, a wealth of data now suggest an active role for ECM in many aspects of biology, from tissue maintenance, regeneration, cell differentiation, to fibrosis and cancer^35,36^.

Given the identification of multiple mesenchymal progenitor markers in the *Tcf21*^+^ population, we next sought to validate expression of these markers in vivo. We took advantage of previously generated *Tcf21*^*mCrem*^ mice, in which a tamoxifen inducible Cre recombinase inserted at the *Tcf21* locus enables the long-term tracing of the descendant populations of the *Tcf21*-expressing cells, whether in the gonads, heart, kidney, and cranial muscle^37^. Specifically, testes were collected from *Tcf21*^*mCrem*^*:R26R*^*tdTom*^ mice after 3 doses of tamoxifen (tdTom^+^ labeled cells referred to as TCF21^lin^), dissociated, stained for a comprehensive panel of mouse MSC markers (SCA1, CD73, CD29, CD34, and THY1) while excluding mature Leydig (cKIT^+^) or immune (CD45^+^) cells, and analyzed using flow cytometry (Supplementary Fig. 1A–F). Since our *Tcf21*^*+*^ cells were initially discovered by enriching SCA1^+^ cells in the testis, we examined the heterogeneity of the SCA1^+^ and TCF21^lin^ cells. As expected, the SCA1^+^ population has a broader representation in the testis than TCF21^lin^ (~3–5% vs. ~1–2%), with about 45% of SCA1^+^ cells being also positive for TCF21^lin^ (Supplementary Fig. 1G)—a proportion consistent with our estimates from scRNA-seq data. Within the SCA1^+^ population, we identified two TCF21^lin^ subpopulations (blue and purple in Supplementary Fig. 1B, C) based on co-expression of MSC markers: a larger TCF21^lin^ population (13.8%, blue) that strongly expressed CD29 (ITGB1), CD73, and CD34, and a smaller discrete TCF21^lin^ population (0.77%, purple) that expressed all MSC markers. In contrast, 80% of the TCF21^lin^ population expresses SCA1 (Supplementary Fig. 1G), and within the TCF21^lin^ populations we identify multiple subtypes that are molecularly heterogenous with respect to MSC marker expression (Supplementary Fig. 1D–F) (see below for the functional assessment of TCF21^lin^ heterogeneity in vitro).

Given the heterogeneity observed within the TCF21^lin^ population, we asked whether TCF21^lin^ subtypes could be further defined by co-expression of previously described interstitial markers including PDGFRA, COUPTFII, CD34, and FGF5, respectively. To answer this question, we co-stained testes from *Tcf21*^*mCrem*^*:R26R*^*tdTom*^ or *Tcf21*^*mCrem*^*:R26R*^*tdTom*^*:PdgfraDGFRA*^*GFP*^ mice with COUPTFII, CD34, and FGF5 antibodies (Supplementary Fig. 1H), but we did not detect a preferred segregation of TCF21^lin^ cells within the PDGRA, COUPTFII, CD34 or FGF5 subpopulation (Supplementary Fig. 1H), suggesting that these populations are heterogeneous on both the cellular and molecular level. Furthermore, for all markers analyzed we find co-stained cells both in the interstitium or surrounding tubules—suggesting that the cellular heterogeneity is not a result of spatial location.

### The TCF21^lin^/SCA1^+^ population has mesenchymal progenitor properties in vitro and can be differentiated to Leydig and myoid cell fates in vitro

Mesenchymal progenitors are typically characterized by their capacity for forming adherent fibroblast-like colonies on plastic (measured as CFU-F: fibroblastic colony-forming units) and for differentiating into adipocytes, osteocytes, and chondrocytes in vitro (reviewed in^38^). Such populations have been isolated from multiple human and mouse organs, including juvenile or adult testes^18,39–41^. To examine whether the adult SCA1^+^ and TCF21^lin^ cells have mesenchymal progenitor-like properties in vitro, we sorted four types of cells: SCA1^−^/cKIT^+^ interstitial cells (control), SCA1^+^/cKIT^−^, SCA1^+^/TCF21^lin^, or TCF21^lin^ cells from *Tcf21*^*mCrem*^*:R26R*^*tdTom*^ animals (Supplementary Fig. 2A) and plated these cells at single-cell density to measure clonogenic potential in vitro. Although SCA1^+^/cKIT^−^, SCA1^+^/TCF21^lin^, and TCF21^lin^ populations all formed colonies in vitro, clonogenic potential differed across populations. The TCF21^lin^ and SCA1^+^/TCF21^lin^ double-positive cells formed the highest number of colonies—~100 colonies per 1000 plated cells, and this was followed by SCA1^+^/cKIT^−^ cells (regardless of the TCF21^lin^ status) and cKIT^+^ cells with ~40 and 10 colonies, respectively (Supplementary Fig. 2B). Altogether, these data demonstrate that SCA1^+^and SCA1^+^/TCF21^lin^ populations have characteristics of mesenchymal progenitors in vitro, but selecting for the TCF21^lin^ population significantly increases colony formation potential.

In addition to expanding in culture, mesenchymal progenitors, under appropriate conditions, can be directed to differentiate into adipocytes, chondrocytes, and osteoblasts. To examine if TCF21^lin^ cells maintain such properties in vitro, we sorted either SCA1^+^/cKIT^−^ or cKIT^+^ cells from *Tcf21*^*mCrem*^*:R26R*^*tdTom*^ animals and examined whether the TCF21^lin^ within the SCA1^+^ population contribute to all three lineages (Supplementary Fig. 2A). Importantly, we found that SCA1^+^ cells robustly differentiated into adipocytes, chondrocytes, and osteocytes as shown by Oil red, Alizarin red, and Alcian blue staining, respectively (Supplementary Fig. 2C). Furthermore, co-immunofluorescence of Osterix (osteocytes), Perilipin (adipocytes), and SOX9 (chondrocytes) with tdTom^+^ (TCF21^lin^) cells demonstrates that the TCF21^lin^ cells within the SCA1^+^ population can contribute to all three lineages in vitro (Supplementary Fig. 2D).

Given the ability of the SCA1^+^/TCF21^lin^ to generate multiple mesenchymal cell types in vitro and the transcriptomic relationship of *Tcf21*^+^ cells with both Leydig and myoid cells (Fig. 1B), we next asked whether the TCF21^lin^/SCA1^+^ population can be directed to differentiate to both Leydig and myoid cells in vitro and if the TCF21^lin^/SCA1^+^ population serves as a multipotent progenitor for Leydig and myoid lineages. To this end, we sorted SCA1^+^/cKIT^−^ (regardless of TCF21 status) or TCF21^lin^/SCA1^+^/cKIT^−^ cells from *Tcf21*^*mCrem*^*:R26R*^*tdTom*^ adult male testes and directed their differentiation to either myoid or Leydig cells using a set of growth factors based on the repertoire of receptors expressed in our scRNA-seq datasets and earlier in vivo genetic findings of Leydig or myoid cell specification (Supplementary Fig. 2E–K)^42–44^. After treating the bulk SCA1^+^ or SCA1^+^/TCF21^lin^ cells with a myoid differentiation cocktail which includes Smoothened agonist (SAG, an activator of Desert Hedgehog), PDGFAA, PDGFBB, ACTIVINA, BMP2, BMP4, and Valproic Acid (outlined in Supplementary Fig. 2E), we observed a morphological conversion of spindle-shaped cells to flattened and striated cells resembling smooth muscle cells (Supplementary Fig. 2F). This conversion was confirmed by expression of smooth muscle cell markers such as smooth muscle actin (Supplementary Fig. 2G, H).

Previous in vivo and in vitro experiments uncovered that Desert Hedgehog (DHH), FGF, and PDGF signaling are stimulatory to Leydig cell differentiation, while Notch signaling and other factors are inhibitory^42–44^. By incorporating these findings from the literature with our scRNA-seq data, we developed a 14-day differentiation protocol that includes PDGFAA, PDGFBB, SAG, FGF2, LiCl_2_, and DAPT (Notch inhibitor) (detailed in Supplementary Fig. 2I). This protocol enables successful differentiation of SCA1^+^/cKIT^−^ or SCA1^+^/TCF21^lin^ cells to Leydig cells (Supplementary Fig. 2J–K). Importantly, the in vitro-derived Leydig cells expressed steroidogenic factor 1 (SF1) (Supplementary Fig. 2K, L) and secreted testosterone (Supplementary Fig. 2M, N). Notably, Leydig cell generation and testosterone secretion was achieved in vitro independent of LH control (Supplementary Fig. 2M, N). Consistent with absence of LH regulation, we detected only low levels of luteinizing hormone/choriogonadotropin receptor (*Lhcgr*) transcripts in day 14 Leydig cells (Supplementary Data 2).

**Fig. 2:**
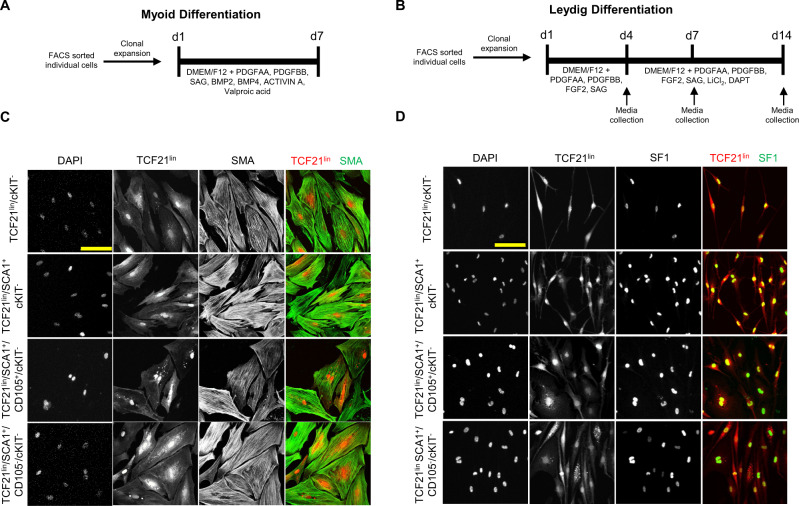
The adult TCF21^lin^ cells are multipotent and can be directed to differentiate to Leydig and smooth muscle cells in vitro. Schematic representation of experimental timelines for myoid (**A**) and Leydig cell (**B**) directed differentiation. Gating strategy used for FACS is presented in Supplementary Fig. 2G. **C** Immunofluorescence staining of smooth muscle actin (SMA) in clonal TCF21^lin^ cells after 7 days of in vitro culture in the presence of differentiation media. Representative images of *n* = 13 technical replicates of TCF21^lin^/cKit^−^, *n* = 6 technical replicates of TCF21^lin^/SCA1^+^/cKit^−^, *n* = 6 technical replicates of TCF21^lin^/SCA1^+^/cKit^−^/CD105^+^, and *n* = 14 technical replicates of TCF21^lin^/SCA1^+^/cKit^−^/CD105. Scale bar: 100 μm for all images in the panel. **D** Immunofluorescence staining of steroidogenic factor 1 (SF1) in clonal TCF21^lin^ cells after 14 days of in vitro culture in the presence of differentiation media. Representative images of *n* = 19 technical replicates of TCF21^lin^/cKit^−^, *n* = 17 technical replicates of TCF21^lin^/SCA1^+^/cKit^−^, *n* = 12 technical replicates of TCF21^lin^/SCA1^+^/cKit^−^/CD105^+^, and *n* = 22 technical replicates of TCF21^lin^/SCA1^+^/cKit^−^/CD105^−^. Scale bar: 100 μm for all images in the panel.

As the SCA1^+^/TCF21^lin^ cells were labeled as a population, the results described above could not distinguish between two scenarios: (1) each TCF21^lin^/SCA1^+^ cell is a multipotent progenitor, capable of adopting any of multiple fates, or (2) these cells are heterogeneous, comprised of multiple subtypes of progenitors that each have been cryptically committed to differentiate into a different somatic lineage. To distinguish between these two scenarios, we sorted individual TCF21^lin^/SCA1^+^ cells into separate wells on 96-well plates and allowed each to expand into individual clones. Following clonal expansion, clones were randomly assigned to either the myoid or Leydig cell differentiation protocol (Fig. 2A, B). Therefore, if these cells have already been primed for one or the other lineage, we would expect that some clones, but not all, that undergo the myoid-inducing treatment will differentiate to the myoid lineage, and likewise some clones in the Leydig treatment group will follow the Leydig lineage. Co-immunostaining with either SMA for myoid cells or SF1 for Leydig cells reveals that all TCF21^lin^/SCA1^+^ clones in either group differentiate to adopt the induced fate 100% of the time (number of clones provided in Supplementary Table 1), suggesting that the TCF21^lin^/SCA1^+^ cells are individually multipotent in vitro (Fig. 2C, D; Supplementary Table 1). However, since the TCF21^lin^/SCA1^+^ population can be further stratified by the expression of CD105 in vivo, we examined whether TCF21^lin^/SCA1^+^ multipotency may be restricted to a specific subset of TCF21^lin^/SCA1^+^ cells. To address this, we sorted and differentiated TCF21^lin^/SCA1^+^/CD105^+^ or TCF21^lin^/SCA1^+^/CD105^−^ cells to Leydig or myoid cells. Consistent with our earlier finding using TCF21^lin^/SCA1^+^ cells, both the TCF21^lin^/SCA1^+^/CD105^+^ and the TCF21^lin^/SCA1^+^/CD105^−^ clonal cells differentiated to both lineages with 100% efficiency (Fig. 2C, D; Supplementary Table 1). These observations suggest that multipotency is not due to heterogeneous subpopulations of TCF21^lin^/SCA1^+^ cells identified by flow cytometry (Supplementary Fig. 1E, F). We caution, however, since these clonal cell experiments were performed in vitro, the multipotency of these cells in vivo remains to be confirmed in future studies.

### Single-cell time-course analysis reveals timing and diverging trajectories during Leydig cell differentiation

**Fig. 3:**
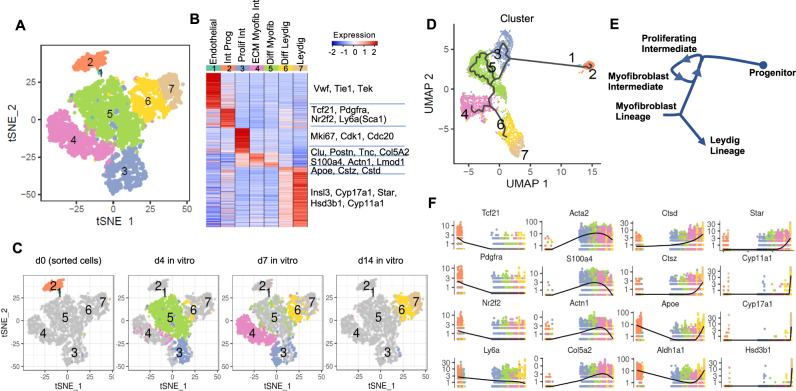
scRNA-seq differentiation trajectory of in vitro derived Leydig cells. **A** Single-cell RNA-seq time course analysis of in vitro Leydig differentiation (days 0, 4, 7, and 14) identifies seven clusters, as visualized in t-SNE space. Note: *n* = 1 replicate per timepoint, but in vitro differentiation was successfully completed 8 times in prior experiments. **B** Heatmap of differentially expressed markers across the seven cluster centroids. **C** Visualization of the contribution of each individual time point at day 0, 4, 7, or 14 to the 7 clusters in t-SNE space. **D** Pseudotime ordering of cells from the 4 time points (days 0, 4, 7, and 14) by Monocle3 in UMAP space, colored by 7 clusters. **E** Schematic annotation of differentiation trajectory as defined by Monocle. **F** Expression profiles of selected markers across the differentiation pseudotime.

We next characterized the Leydig cell differentiation process in vitro using scRNA-seq. To this end, we collected and analyzed ~6500 cells across four time-points along the in vitro differentiation process (d0 sorted SCA1^+^/cKIT^−^, d4, d7, and d14 in culture). By clustering the merged dataset and using gene expression and marker gene analysis we identified seven distinct clusters (Fig. 3A, B). When overlaying time points on the different clusters, we find that freshly sorted SCA1^+^/cKIT^−^ cells at d0 contribute to clusters 1 and 2 (Fig. 3C). A small number of cells in cluster 1 express von Willebrand factor (*Vwf*) and the receptor tyrosine kinase *Tie-1*, indicating that some endothelial cells also express *Sca1* (Fig. 1C, 3B; Supplementary Data 2). However, cluster 2, which constitutes the majority of SCA1^+^/cKIT^−^ cells, is the interstitial progenitor population that expresses *Tcf21*, *Pdgfra*, and *CoupTFII* (Fig. 3B; Supplementary Data 2).

Four days after exposure to expansion media, cells dominate in clusters 3 and 5 with a smaller number of cells appearing in clusters 4 and 6 (Fig. 3C). Cells in cluster 3 are actively proliferating, as reflected by expression of proliferative marker *Mki67*, cyclins *Cdk1* and *Cdc20*, and mitotic microtubule associated proteins (Fig. 3B; Supplementary Data 2). Cells in cluster 5 are postmitotic myofibroblasts expressing genes involved in actin cytoskeleton dynamics and remodeling (e.g. *S100a4*, *Actn1*, *Lmod1*, *Nexn*, *Acta2*) (Fig. 3B; Supplementary Data 2). Three days (d7) after transition to differentiation media, cells have become directed to an ECM-depositing myofibroblast cell state (expressing: *Clu*, *Postn*, *Tnc*, *Col5A2*; cluster 4) or a progenitor Leydig cell state (cluster 6) (Fig. 3B, C; Supplementary Data 2). Although cells in cluster 4 do not ultimately contribute to Leydig differentiation, they express fibroblast markers (*Col5a2*, *Postn*, *Tnc*) as well as extracellular-matrix related proteins involved in tissue remodeling (Fig. 3B, C; Supplementary Data 2). Interestingly, this population also expresses *Pdgfa*, raising the possibility that cluster 4 cells serve as an intermediate supportive cell population required to promote continued differentiation of Leydig cells.

By day 14 (10 days of exposure to differentiation media), cells in clusters 6 and 7 have become more differentiated (Fig. 3C). Cells in cluster 6 appear to prepare for steroidogenesis by increasing expression of lysosome/exosome genes, likely employing autophagy to degrade cellular components into steroid building blocks like cholesterol and fats (Fig. 3B; Supplementary Data 2). Previously, autophagy in Leydig cells was shown to be a rate-limiting step for testosterone synthesis^45^. Apolipoprotein E (*ApoE*) is also expressed at this time, indicating that LDL uptake is occurring which is critical for steroidogenesis, in line with genetic evidence in *ApoE/Ldlr* knockout mice^46^. Finally, in cluster 7, steroidogenic enzymes *Cyp17A1*, *Hsd3B1*, *StAR*, *Cyp11A1* are expressed, as well as the mature Leydig cell factor *Insl3*, indicating a cellular state with functional steroidogenesis (Fig. 3B, F; Supplementary Data 2). The in vitro developmental progression based on time point sampling also confirmed Monocle3 pseudotime analysis (Fig. 3D–F), where sorted progenitors give rise to a cycle of proliferating and differentiating intermediates. Cells then branch into two differentiation trajectories, one aborting in cluster 4, which is an ECM producing myofibroblast, possibly a support intermediate cell, while the remaining cells proceed through clusters 6 and 7 which lead to differentiated Leydig cells (Fig. 3D–F).

Given our success with generating molecularly functional (testosterone secreting) Leydig cells in vitro, we next asked whether the in vitro derived Leydig cells bear resemblance to in vivo Leydig cells by comparing to previously published adult and fetal somatic cell states^13,33^. Notably, the in vitro intermediate states (clusters 2–5) in Leydig cell differentiation correlate with early interstitial progenitors in the fetal gonad, whereas clusters 6 and 7 have a higher correlation to fetal Leydig cells (Supplementary Fig. 3A). When comparing to the adult testis, the in vitro intermediate states (Clusters 2–5) correlate more closely with the *Tcf21*-expressing interstitial population, whereas clusters 6 and 7 have the highest correlation to adult Leydig cells (Supplementary Fig. 3B). Interestingly, overall the in vitro derived Leydig cells have higher correlation to adult Leydig cells than fetal Leydig cells (*r* = 0.84 vs. 0.58, respectively) (Supplementary Fig. 3A, B).

### TCF21^lin^ cells contribute to somatic lineages in the male gonad in vivo

**Fig. 4:**
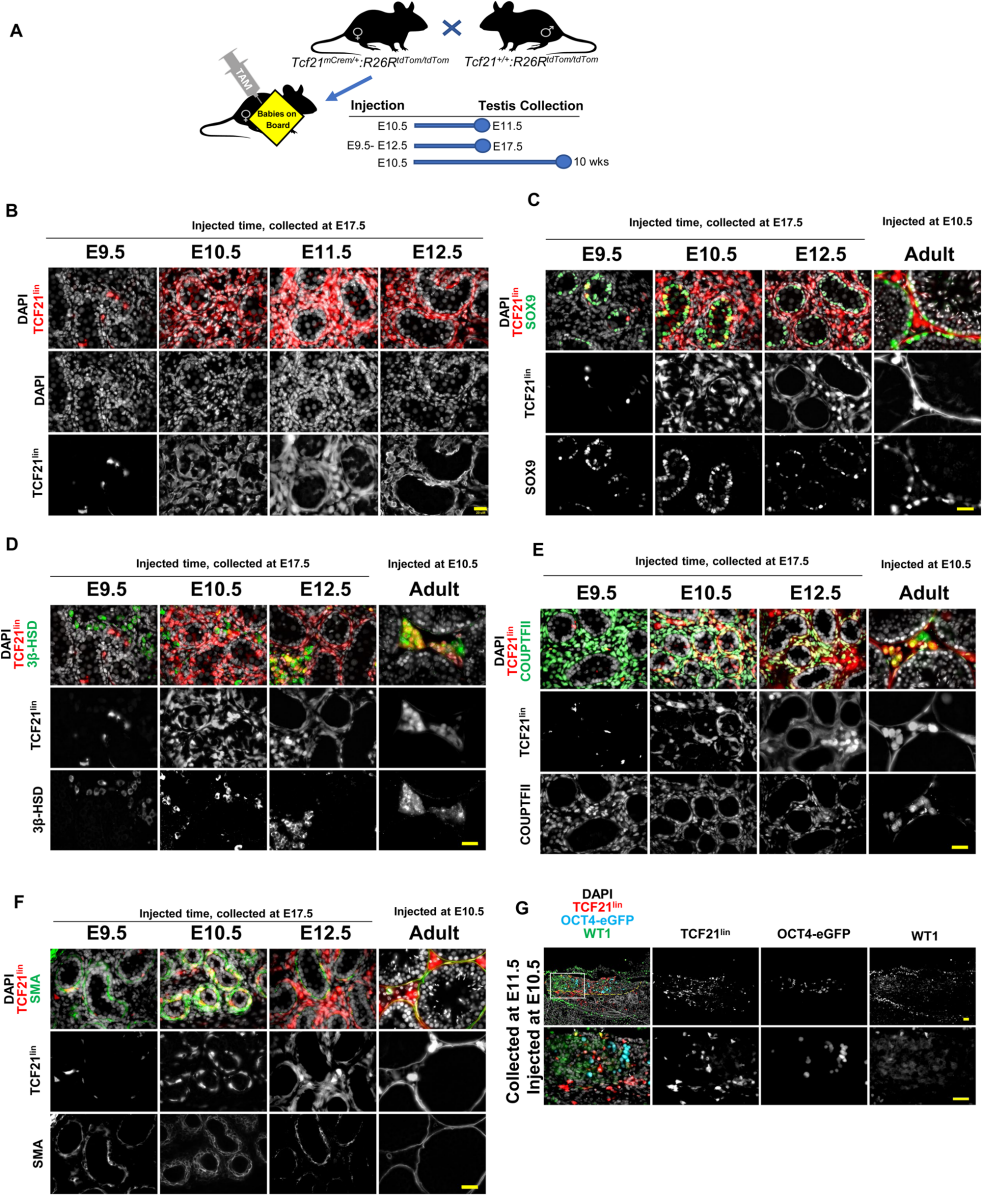
The TCF21^lin^ population contributes to multiple somatic lineages in the fetal and adult testis. **A** Experimental timeline used for *Tcf21* lineage tracing analysis. *Tcf21*^*mCrem*^*:R26R*^*tdTom*^ timed pregnant females were injected with a single dose of Tamoxifen at E9.5, 10.5, E11.5 or 12.5 and testes were analyzed at E17.5 or 10 weeks. **B** The TCF21^lin^ cells at E10.5 (*n* = 17) and E11.5 (*n* = 11) contribute to all major somatic cell populations in the fetal gonad, whereas the E12.5 (*n* = 11) TCF21^lin^ cells give rise only to testis interstitial cells. **C**–**F** Co-immunostaining of fetal or fostered adult TCF21^lin^ testis cross-sections with Sertoli cell marker SOX9 (green; **C**, *n* = 5 for E9.5 and E12.5, *n* = 3 for E10.5), Leydig cell marker 3β-HSD (green; **D**, *n* = 5 for E9.5, *n* = 3 for E10.5 and E12.5), interstitial cell marker COUPTFII (NR2F2; green in **E**, *n* = 5 for E9.5, E10.5, and E12.5), and a myoid cell marker alpha smooth muscle actin (SMA, green in **F**, *n* = 5 for E9.5, *n* = 3 for E10.5, *n* = 4 for E12.5). **G** The TCF21^lin^ in the fetal testis of *Tcf21*^*mCrem*^*; R26R*^*tdTom*^; *Oct4-eGFP* embryos are present in both the coelomic epithelium and mesonephros. Colocalization of WT1^+^ (Green) cells in the E11.5 gonad with TCF21^lin^ cells (*n* = 2). In all panels the nuclear counterstain is DAPI (white **B**–**G**, *n* = 2). Scale bars for all **B**–**G**: 20 μm.

Given our ability to differentiate TCF21^lin^ cells to Leydig or myoid cells in vitro, we next asked if the *Tcf21* lineage can serve as a somatic progenitor in vivo. To this end, we performed lineage-tracing from early developmental time points in *Tcf21*^*mCrem*^*:R26R*^*tdTom*^ mice and analyzed fully formed testes at E17.5 and adult testes at 10 weeks. Specifically, timed pregnant females were given a single dose of tamoxifen at either gestational days E9.5, E10.5, E11.5 or E12.5 (Fig. 4A). We verified that TCF21^lin^ labeling was absent in embryonic gonads harvested from vehicle-treated timed pregnant females, confirming tight regulation of the tamoxifen inducible Cre (Supplementary Fig. 4A). Additionally, lineage traced cells did not overlap with Vasa, a germ cell marker, confirming specificity of the *Tcf21*^*mCrem*^ line (Supplementary Fig. 4B).

Immunofluorescence and histological analysis of E17.5 male gonads revealed marked differences in the extent of labeling across the different tamoxifen injection time points (Fig. 4B). We observed relatively fewer TCF21^lin^ cells from E9.5 injected animals, yet those cells co-localized with multiple somatic lineages, as evidenced by colocalization with markers for Sertoli cells (SOX9), interstitial cells (COUPTFII) and, to a lower extent, fetal Leydig cells (3BHSD), and myoid (SMA) cells (Fig. 4C–F, Supplementary Fig. 4C–F). In contrast, a greater number of TCF21^lin^ cells are observed in the E17.5 gonads collected from animals treated with tamoxifen at E10.5, E11.5, and E12.5, possibly due to broader expression of *Tcf21* in multiple somatic progenitors at these later injection timepoints (Fig. 4C–F, Supplementary Fig. 4C–F). The TCF21^lin^ cells at these different timepoints again contributed to Sertoli (SOX9), fetal Leydig (3BHSD), interstitial (COUPTFII, PDGFRA, and GLI), and myoid (SMA) cells (Fig. 4C–F, Supplementary Fig. 4C–H). Curiously, a significant fraction of E9.5 or E10.5 TCF21^lin^ cells contribute to Sertoli cells, but these cells account for only a fraction of all SOX9^+^ cells in the fetal and postnatal testis (Supplementary Fig. 4C). This suggests either incomplete labeling of TCF21^lin^ cells or Sertoli cells arising from multiple somatic progenitor populations.

To determine the origin of TCF21^lin+^ cells in the embryonic gonad and potential overlap with the WT1^+^ population—a somatic progenitor previously shown to give rise to Sertoli and interstitial populations including adult Leydig cells^47^—we injected timed pregnant *Tcf21*^*mCrem*^*:R26R*^*tdTom*^*;Oct4-eGFP* females with a single dose of tamoxifen at E10.5 and collected and stained whole mount gonads with WT1 at E11.5. In the E11.5 gonads, the TCF21^lin^ cells localize both to the coelomic epithelium and the mesonephros, making it difficult to determine if TCF21^lin^ cells truly originate from the coelomic epithelium or mesonephros, or are present in either location. However, we find that TCF21^lin^ cells partially overlap with the WT1^+^ cells in the coelomic epithelium, but many cells are either TCF21^lin^ or WT1^+^, suggesting these are possibly two distinct populations (Fig. 4G).

Unlike most somatic cell populations in the testis, the steroid-producing Leydig cells are unique in that they arise in two distinct waves. To ascertain whether the fetal-derived TCF21^lin^ cells persist in the postnatal testis and give rise to adult Leydig cells, *Tcf21*^*mCrem*^*:R26R*^*tdTom*^ time pregnant females received a single injection of tamoxifen at E10.5 and the pups were fostered and matured to adulthood (see Methods; Fig. 4A). In 10-week-old male testes, we found that a fraction of adult Sertoli, peritubular myoid, endothelial cells, and interstitial cells are tdTom^+^ (i.e., derived from TCF21^lin^) (Fig. 4C–F). Furthermore, we observe overlap between TCF21^lin^ and 3BHSD in the adult testis, suggesting that the fetal TCF21^lin^ population gives rise to adult Leydig cells (Fig. 4D). Taken together, we demonstrate that the fetal TCF21^lin^ contributes to all adult somatic lineages of the testis. However, since the overlap is incomplete in all somatic populations this raises two possibilities: incomplete labeling or an alternative progenitor source population.

Although the fetal TCF21^lin^ cells in vivo contribute to multiple somatic progenitor populations, the limitations of labeling a single somatic progenitor in the fetal testis has hampered our ability to conclusively determine whether the fetal TCF21 population is a heterogeneous population already committed to different fates, or if each cell is individually multipotent.

### The TCF21^lin^ gives rise to multiple fetal and adult ovarian somatic cell types

Prior to sex determination in mammals, the gonadal primordium is bipotential, meaning that the gonadal mesenchyme has the ability to give rise to either male or female support cell and steroidogenic cell lineages^32^. Once *Sry* expression is turned on during a critical window of fetal development, testis differentiation is initiated^48^. Despite an early commitment to either male or female somatic cell types, genetic studies in mouse have shown that terminally differentiated cell types must be actively maintained throughout life. For example, loss of either *Dmrt1* in Sertoli cells or *Foxl2* in granulosa cells can trigger reciprocal cell fate conversions (from Sertoli to granulosa cell fate or granulosa to Sertoli cell fate, respectively)^49–51^. Taking into account the known gonadal mesenchyme plasticity and the ability of TCF21^lin^ cells to give rise to multiple somatic lineages in the testis, we next asked if TCF21^lin^ cells are present in the fetal ovary and whether they might give rise to analogous cell types in females. For our female gonad experiments, we injected timed pregnant female *Tcf21*^*mCrem*^*:R26R*^*tdTom*^*;Oct4-eGFP* mice with a single dose of tamoxifen at E10.5 and collected female embryonic gonads at E11.5. Our analysis shows that TCF21^lin^ cells are present in the coelomic epithelium and mesonephros, similarly to what we had observed in male embryonic gonads (Supplementary Fig. 4K). We then injected tamoxifen in timed-pregnant females at E10.5, E11.5, or E12.5 *Tcf21*^*mCrem*^*:R26R*^*tdTom*^ mice and found broad somatic cell labeling in the female gonads at E17.5 (Supplementary Fig. 4L). Co-staining ovarian cross-sections with terminally differentiated markers shows that TCF21^lin^ cells overlap with markers for granulosa cells (FOXL2^+^), interstitial cells (COUPTFII^+^), and smooth muscle cells (SMA^+^), and does not overlap with the germ cell markers VASA or OCT4 (Supplementary Fig. 4M–P).

In the postnatal mouse ovary, the fetal TCF21^lin^ population contributes to multiple adult somatic lineages including granulosa cells (FOXL2^+^ and WT1^+^) of primordial and growing follicles, and endothelial cells (PECAM^+^), but not germ cells (Supplementary Fig. 4Q). Furthermore, we find that the TCF21^lin^ cells surround the follicles and co-expresses the theca cell marker 3BHSD^+^^52^. Previous studies have shown that theca cells are derived postnatally from GLI1^+^ and/or WT1^+^ populations^53,54^. Therefore, we asked if the fetal TCF21^lin^ cells co-express WT1. To this end, we examined E11.5 embryonic gonads from timed pregnant *Tcf21*^*mCrem*^*:R26R*^*tdTom*^*;Oct4-eGFP* mice and co-stained gonad sections for WT1 (Supplementary Fig. 4K). WT1 and TCF21^lin^ appear to be largely in distinct populations with the exception of a few cells in the coelomic epithelium (Supplementary Fig. 4K). Therefore, the fetal TCF21^lin^ population is largely distinct from the WT1 population, yet, it contributes to all somatic lineages in the adult ovary including: granulosa cells (FOXL2^+^ and WT1^+^) of primordial and growing follicles, endothelial cells (PECAM^+^), and theca cells (3BHSD^+^), suggesting that the female gonad, like the male gonad, may have multiple somatic progenitors.

### Tcf21^lin^ cells regenerate Leydig cells in the adult testis in response to chemical ablation

Although somatic cells of the testis are considered to be postmitotic and do not naturally turnover, our steady state scRNA-seq datasets identified rare *Tcf21*^+^ cells that express low levels of either steroidogenic acute regulatory protein (*StAR*) or *Sma*, suggesting that a rare subset of Tcf21^+^ cells transition to either Leydig or myoid cells, respectively. We then examined if the adult TCF21^lin^ is capable of serving as a somatic progenitor at least for adult Leydig cells in the testis. Specifically, animals were treated with EDS to reduce Leydig cell numbers and the testes were collected and assessed to determine (1) if regeneration does occur and (2) if TCF21^lin^ contributes to the regeneration.

EDS treatment was previously used in rats to selectively ablate Leydig cells^55–58^. Although there is strong species specificity and animal-to-animal variation in Leydig cell sensitivity to EDS^59^, two 300 mg/kg EDS injections in C57BL/6 mice spaced 48 h apart (Supplementary Fig. 5A) resulted in a reduction of mature Leydig cells in the adult testis as evidenced by cell death and CYP17A1 protein levels (Supplementary Fig. 5B, C). At 12 h post final injection (hpfi), we detected TUNEL positive Leydig cells, but given the spacing of 48 h between the first and second injection, the majority of apoptotic events likely preceded the time of analysis and could therefore not be quantified (Supplementary Fig. 5B). However, consistent with Leydig cell loss, we find that CYP17A1 protein expression decreased as early as 12 hpfi and had largely recovered by 14 dpfi (Supplementary Fig. 5C). We confirmed that the recovery of CYP17A1 protein expression was not simply due to Leydig cell hypertrophy, as the Leydig cell diameter is similar between the EDS- and vehicle-treated animals at 14 dpfi (Supplementary Fig. 5D).

**Fig. 5:**
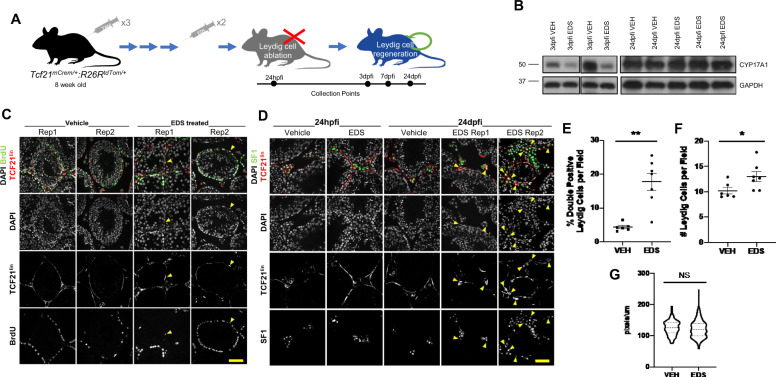
The TCF21^lin^ population regenerates Leydig cells in vivo after injury. **A** Schematic representation of the experimental method used to ablate Leydig cells in the *Tcf21*^*mCrem*^*:R26R*^*tdTom*^ background. **B** Representative CYP17A1 protein immunoblots from EDS- and vehicle-treated animals at 3 dpfi and 24 dpfi. A total of *n* = 8 independent experiments were performed. **C** Representative images of BrdU^+^ cells in the testis of vehicle- and EDS-treated animals (representative from *n* = 2 vehicle, *n* = 3 EDS). **D** Colocalization of TCF21^lin^ (tdTom) and Leydig cell marker SF1 in EDS- or vehicle-treated *Tcf21*^*mCrem*^*:R26R*^*tdTom*^ animals at 24 h post final injection (24 hpfi) or 24 days post final injection (24 dpfi). Representative images from *n* = 6 vehicle, *n* = 7 EDS animals. **E** Quantification of the percentage of Leydig cells expressing both SF1 and TCF21^lin^ (tdTom) per field at 24 dpfi (Note—each circle represents the percentage per animal. A total of *n* = 6 vehicle, *n* = 7 EDS were analyzed). Data are presented as mean ± SEM. *P* = 0.0018. **F** Quantification of Leydig cell number per field of view at 24 dpfi (*n* = 6 vehicle, *n* = 7 EDS). Data are presented as mean ± SEM. *P* = 0.042. **G** Average Leydig cell diameter measurements in *Tcf21*^*mCrem*^*:R26R*^*tdTom*^ EDS or vehicle-injected animals, after Leydig cell recovery at 24 dpfi (*n* = 3 per condition). Lines indicate mean and quartiles. *P* = 0.092. All statistical tests were performed using Welch’s unpaired, two-sided *t*-tests. Scale bars: 20 μm for all images in **C**, **D**.

To determine if regenerating Leydig cells were derived from the adult testis TCF21^lin^ we injected 8-week-old *Tcf21*^*mCrem*^*:R26R*^*tdTom*^ mice with three 2 mg tamoxifen injections, and then treated the mice with two 300 mg/kg EDS injections spaced 48 h apart (Fig. 5A). Similar to EDS-treated C57BL/6 animals, CYP17A1 protein levels in *Tcf21*^*mCrem*^*:R26R*^*tdTom*^ EDS-treated animals decreased at 3 dpfi and recovered by 24 dpfi (Fig. 5B). To ensure that the reduction in CYP17A1 is due to Leydig cell loss, we quantified the number of SF1^+^ cells in EDS- and vehicle-treated animals at 3 dpfi. In 3 dpfi animals, ~20% of all cells in testis cross-sections are SF1^+^ in the vehicle-treated animal, whereas the number of SF1^+^ cells is reduced to 5% in the EDS-treated animals, suggesting many Leydig cells were lost (*n* = 3 vehicle and *n* = 3 EDS animals). To test whether the TCF21^lin^ population contributes to Leydig cell regeneration, we examined if TCF21^lin^ cells proliferate in response to injury and contribute to the regenerated Leydig cells. At 3 dpfi, TCF21^lin^ cells surrounding the tubules re-entered the cell cycle as detected by colocalization of BrdU and TCF21^lin^ (Fig. 5C). Importantly, by 24 dpfi in EDS-treated animals, ~18% of SF1^+^ Leydig cells are Tcf21^lin^ positive as compared to ~5% in the vehicle-treated animals (Fig. 5D, E). Furthermore, we find that the EDS-treated animals have a higher number of SF1^+^ cells as compared to controls (Fig. 5F), which is consistent with an overcompensation in CYP17A1 protein levels observed in EDS-treated animals (Fig. 5B) and the absence of Leydig cell hypertrophy (Fig. 5G).

To independently validate the role of TCF21^lin^ cells in testis somatic cell regeneration, we performed allogenic transplants. Specifically, we sorted adult SCA1^+^ cells from *Tcf21*^*mCrem*^*:R26R*^*tdTom*^ animals and transplanted them into the testis interstitium of EDS-treated C57BL/6 animals where no Leydig cells in the host animal will be TdTom^+^ (Supplementary Fig. 5E). We reasoned that if the transplanted TCF21^lin^ population contributed to Leydig cell regeneration, then we should detect TCF21^lin^/SF1 double-positive cells in the C57BL/6 EDS-treated animal. By 24 hpfi, we found TCF21^lin^ cells homed to the basement membrane, and by 7 dpfi, we began detecting TCF21^lin^/SF1^+^ cells (Supplementary Fig. 5F), suggesting that the Tcf21^lin^ cells engrafted in the ablated C57BL/6 mouse testis and gave rise to SF1^+^ cells.

Therefore, by using the *Tcf21*^*mCrem*^*:R26R*^*tdTom*^ EDS model and the TCF21^lin^ transplant approach in EDS-treated C57BL/6 mice, we demonstrate that the adult Tcf21^lin^ population in the testis can at least serve as a reserve Leydig progenitor in response to Leydig cell loss.

### Peritubular myoid cells of the testis can regenerate after injury in adult testis

While we demonstrated that Leydig cells can be regenerated in response to tissue injury, it is unclear whether additional somatic cells in the testis can do the same. Previous studies have shown that Sertoli cells can be replaced by transplantation but cannot be regenerated following targeted diphtheria toxin (DTX) treatment^60–62^, but the regenerative ability of peritubular myoid cells has not been assessed. To examine whether (1) peritubular myoid cells regenerate and (2) if TCF21^lin^ cells contribute to the regeneration, we treated 6–13-week-old *Myh11*^*cre-eGFP*^*; Rosa26*^*iDTR/+*^ mice (referred to as *MYH11-cre:iDTR* hereafter) with multiple low doses of DTX to balance animal survival and myoid cell ablation and collected testes at 12 hpfi and 4 dpfi (Supplementary Fig. 6A). By 12 hpfi, TUNEL positive cells were present on the tubule basement membrane in *Myh11*^*cre-eGFP*^*; Rosa26*^*iDTR/+*^ mice but absent in control mice but were no longer detectable by 4 dpfi (Supplementary Fig. 6B). However, at 4 dpfi the testis cross-sections of *Myh11*^*cre-eGFP*^*; Rosa26*^*iDTR/+*^ animals continue to display vacuoles and disordered tubules, whereas, these histological features were absent from controls (Supplementary Fig. 6C). By 4 dpfi, we detect BrdU positive smooth muscle cells surrounding the basement membrane (Supplementary Fig. 6D, yellow arrow) as well as BrdU positive cells on the tubule surface which lacked SMA expression (Supplementary Fig. 6D, white arrow), indicating both neighboring peritubular smooth muscle cells and nearby interstitial cell progenitors re-enter the cell cycle to regenerate the basement membrane in the DTX-treated *Myh11*^*cre-eGFP*^*; Rosa26*^*iDTR/+*^mice. However, since our TCF21 antibody did not yield clear immunofluorescence staining, it could not be determined whether proliferating cells are TCF21^+^.

In an attempt to overcome these limitations, we sorted SCA1^+^/TCF21^lin^ cells from *Tcf21*^*mCrem*^*:R26R*^*tdTom*^ animals and transplanted these cells into the interstitial space of *Myh11*^*cre-eGFP*^*; Rosa26*^*iDTR/+*^ DTX-treated animals. Within 24 h after transplant, we were able to detect TCF21^lin^ cells surrounding the damaged tubules (Supplementary Fig. 6E, F), but failed to observe any TCF21^lin^ become SMA^+^ at this time point (Supplementary Fig. 6G). Therefore, unlike with Leydig cells, for which we relied on EDS to induce cell-specific ablation, our effort to ablate myoid cells relied on the use of *Myh11*^*cre-egfp*^*; Rosa26*^*iDTR/+*^ which is expressed in multiple organs, leading to lower animal viability and preventing the analysis of the fate of TCF21^lin^ transplanted cells at later time-points.

In summary, we have demonstrated that peritubular myoid cells can be regenerated. Unlike in the case of Leydig cells described above, whether myoid cells can be derived from TCF21^lin^ cells could not be ascertained at this time and will require additional tools.

### Adult TCF21^lin^ cells contribute to somatic turnover in the testis during natural aging

**Fig. 6:**
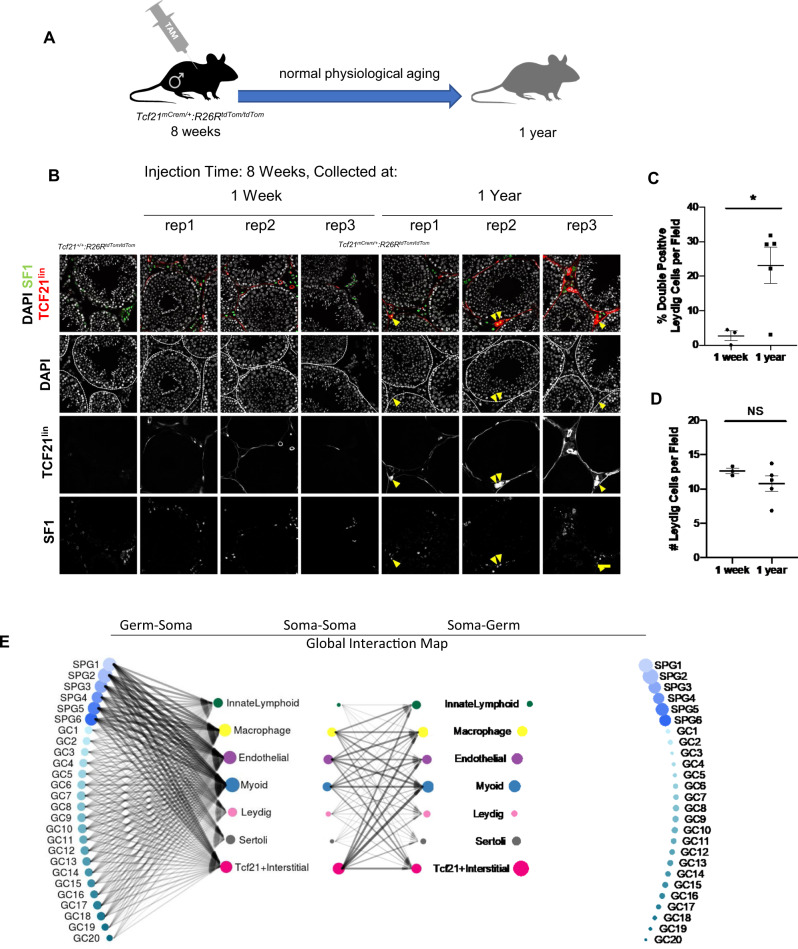
The TCF21^lin^ population maintains testis tissue homeostasis during aging. **A** Schematic representation of TCF21 lineage tracing in the aging testis. **B** Colocalization of TCF21^lin^ (tdTom) and Leydig cell marker SF1 (green) at 1 week or 1 year following a single injection of tamoxifen in 8-week-old adults. Scale bar: 20 μm all panels. DAPI is used as the nuclear counterstain (white). (Oil only control *n* = 2, demonstrating the tight control of TCF21-Cre, *n* = 3 for 1 week, *n* = 5 for 1 year). **C** Quantification of the percent of Leydig cells expressing both SF1 and TCF21^lin^ (tdTom) per field (Note—each circle/square represents the percentage per animal; *n* = 3 for 1 week, *n* = 5 for 1 year). *P* = 0.016. Data are presented as mean ± SEM. All statistical tests were performed using Welch’s unpaired, two-sided *t*-tests. **D** Quantification of Leydig cell number per field (Note—each circle/square represents the percentage per animal; *n* = 3 for 1 week, *n* = 5 for 1 year). *P* = 0.19. Data are presented as mean ± SEM. All statistical tests were performed using Welch’s unpaired, two-sided *t*-tests. **E** Summary of putative ligand–receptor interactions in the mouse testis between the germline and soma (left), within soma (center), and soma and germline (right). Arrows summarize top 5% of all interactions. ScRNA-seq data from^42^, processed as described in^34^. Symbol size indicates the number of receptor–ligand interactions contributed by a cell type and line width shows the number of interactions between the two cell types.

Once established, the somatic cells of the testis are maintained throughout a male’s reproductive age. However, a study in rats using [3H]thymidine labeling to detect proliferation suggested that Leydig and possibly peritubular myoid cells may undergo rare events of cellular turnover during an animal’s natural lifespan^63^. We then asked to what extent adult mouse testis somatic cells turnover during natural aging, and secondly, whether new cells would be derived from TCF21^lin^ progenitors. To answer this question, we performed a long-term lineage tracing experiment where 8-week-old (adult) *Tcf21*^*mCrem*^*:R26R*^*tdTom*^ animals were injected with a single dose of 2 mg tamoxifen. Animals were euthanized either 1-week past final injection or 1-year past final injection (aged mice) (Fig. 6A). In animals with 1-week labeling we detected rare TCF21^lin^ cells surrounding the basement membrane, and these cells do not significantly overlap with Leydig cell markers such as SF1 (Fig. 6B). In aged mice, we detected a significant increase inTCF21^lin^ and SF1 double-positive cells (Fig. 6C) as well as more labeling around peritubular cells suggesting that peritubular cells may also be replenished (Fig. 6B). This extent of labeling varied by individual animal which could be due to tamoxifen injection efficiency or true natural biological variability in inbred mice. Interestingly, while there is no significant difference in Leydig cell number between short-term labeled and aged individuals, there is more variability among aged individuals (Fig. 6D). Nevertheless, these data indicate that somatic cells of the testis naturally turnover, possibly at low rates, and the TCF21^lin^ population contributes to the replenishment and maintenance of the adult Leydig cell population, and likely peritubular myoid cells as well.

### Additional intercellular interactions suggested by scRNA-seq data

To gain a sense of cellular crosstalk between *Tcf21*^*+*^ cells and germ/somatic cells, we focused on previously documented ligand–receptor (L–R) pairs that are highly variable among major cell types in our scRNA-seq data, and calculated Interaction Scores between germ cells and somatic cells, or among somatic cells of the testis (Fig. 6E, Supplementary Data 3). We previously showed that the *Tcf21*^+^ population has more potential interactions with spermatogonial populations than other germ cells^34^ (Fig. 6E, right panel), Similarly, we find that spermatogonia can potentially signal back to the *Tcf21*^+^ population (Fig. 6E, left panel) via PDGFA, various ADAMs, calmodulins, FGFs, and guanine nucleotide binding proteins (Supplementary Data 3).

Within the somatic compartment, *Tcf21*^*+*^ cells have the greatest potential interactions with endothelial, myoid, and macrophages (Fig. 6E, middle panel) involving receptor–ligand signaling systems such as *Lrp1* (*Cd91*), a multifunctional, endocytic receptor capable of binding a vast array of ligands^64^ and known to regulate the levels of signaling molecules by endocytosis, as well as directly participate in signaling for cell migration, proliferation, and vascular permeability (reviewed in^65^). Like other tissue mesenchymal progenitors, *Tcf21*^+^ cells may also modulate local inflammatory responses, as they express Thrombomodulin (*Tbhd*) which interacts with and can proteolytically cleave the pro-inflammatory molecule *Hmgb1* (reviewed in^66^). Additionally, there are several more cell-specific interactions with myoid cells (*Tgfb2-Tgfbr3*;*Gpc3-Cd81*), endothelial cells (*Cxcl12-Itgb1*; *Pdgfa-Pdgfra*; and *Vegfa-Itgb1*), and macrophages (*Igf1-Igf1r; F13a1-Itgb1*) (Supplementary Data 3). Several of these putative interactions are involved in growth, wound healing, phagocytosis, and matrix remodeling in various mesenchymal cell types^67^. These putative interactions will need to be validated by spatial analysis and/or functional perturbations of individual signaling pathways.

### The *Tcf21*^+^ population in the testis resembles resident fibroblast populations in other tissues

**Fig. 7:**
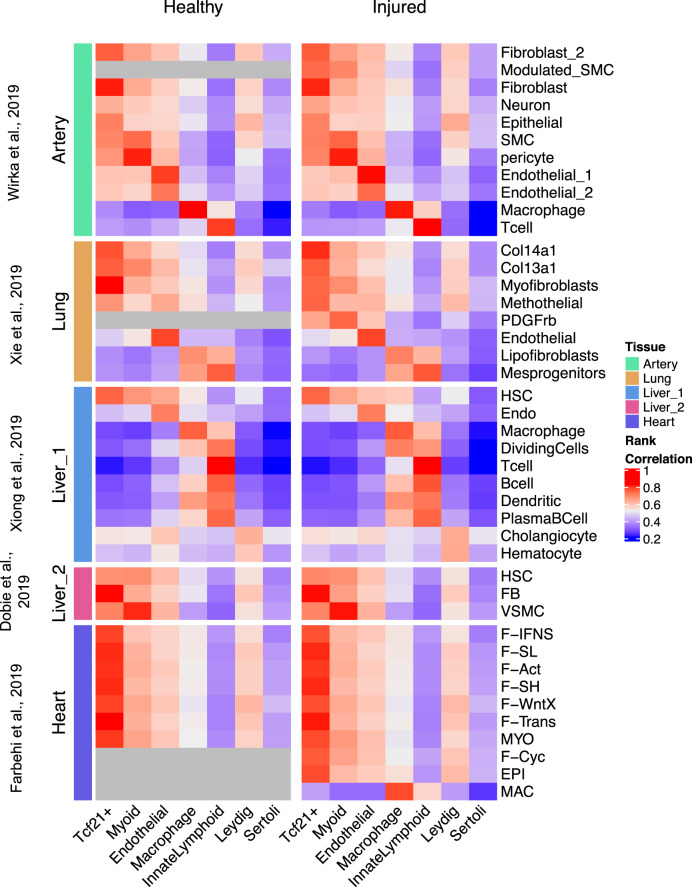
The adult *Tcf21*^+^ interstitial population resembles fibroblasts in other tissues. **A** Rank correlation of scRNA-seq cluster centroids of somatic cells from wild-type adult mouse testes with other tissues in healthy adult mice (left panels) or after injury (right panels). Gray blocks indicate cell types not present in healthy datasets.

Finally, given the essential role of *Tcf21*^+^ cells in mesenchymal development of many tissues, including the heart, lung, and kidney^27,29,37,68^, we sought to understand whether the adult testis *Tcf21*^+^ population resembles other *Tcf21*^+^ mesenchymal populations found in single-cell analyses of other tissues. Our comparison of testis somatic cells with the publicly available scRNA-seq datasets from coronary artery, heart, lung, and liver^69–73^ find that the testis *Tcf21*^+^ population most closely resembled the resident fibroblast or myofibroblast populations (Fig. 7, left), as well as the fibroblast/myofibroblast populations that appear transiently after tissue injury (Fig. 7, right). These diverse fibroblast cell types have been documented across tissues and implicated in fibrotic damage and tissue regeneration, even when they may differ with respect to cellular markers or nomenclature (reviewed in^74^). Our results indicate that they are transcriptomically similar to the testicular *Tcf21*^+^ population characterized here, suggesting that they collectively represent an emerging class of resident adult progenitor cells playing similar roles in tissue maintenance and repair across multiple organ systems.

## Discussion

Tcf21 is a basic helix-loop-helix transcription factor, known to have roles in the development of numerous organs, including the testis^27–29^. During testis development, *Tcf21* is expressed in the bipotential gonadal ridge at E10.5 similar to other key transcription factors, including WT1 and GATA4^75^, and loss of Tcf21 results in gonadal dysgenesis^29,30^. Here, our lineage tracing data show that the fetal TCF21^lin^ population is a bipotential gonadal progenitor giving rise to most somatic cell types including steroid-producing cells (fetal and adult), stromal/interstitial cells, and supporting cells, as well as vasculature, consistent with a common bipotential progenitor model recently described^32,33^. Gonadal organogenesis is a complex process with multiple somatic progenitors have been described in both males and females including: WT1, COUPTFII, NESTIN, and PDGFRA^18,21,23,47,76^. To a certain extent, our data reconcile these findings by showing that TCF21^lin^ overlaps with many of these markers, but our data supports a multi-progenitor model for both interstitial and Sertoli cell populations that are molecularly and cellularly heterogeneous.

Furthermore, we demonstrate that the TCF21^lin^ cells share characteristics with adult mesenchymal progenitors (MPs) for example: TCF21^lin^/SCA1^+^cells can self-renew in vitro and possess numerous traits like homing ability, secretion of molecules with anti-inflammatory and immunoregulatory effects, and have multi-lineage potential^77,78^. Furthermore, we show that the SCA1^+^/TCF21^lin^ cells can be directed to differentiate to myoid and Leydig cells in vitro. Importantly, the in vitro derived Leydig cells secrete testosterone and highly resemble in vivo*-*derived Leydig cells. The availability of such a robust differentiation protocol makes it possible to use in vitro-derived cells to study how environmental toxicants effect steroidogenesis or Leydig cell function/biology.

Testosterone is essential for the development and maintenance of male characteristics and fertility. Reduced serum testosterone affects millions of men and is associated with numerous pathologies including infertility, cardiovascular diseases, metabolic syndrome, and decreased sexual function. Although exogenous replacement therapies are largely successful in ameliorating these symptoms, they carry increased risks of cardiovascular and prostate disease or infertility^79–81^. Therefore, identifying a progenitor population and/or natural mechanism to restore testosterone levels in vivo and combat hypogonadism or age-related decline in testosterone levels is critical^82,83^. Here, we show that resident TCF21^lin^ cells or TCF21^lin^ allogenic transplants can be activated to support Leydig cell regeneration and replenish Leydig cells upon injury or aging. To date there have been several putative stem Leydig cell populations described in the fetal and early postnatal testis of multiple species. These likely analogous populations are demarcated by diverse cell surface markers that are often species specific, like CD90 for rat and CD51 for mouse (reviewed in^82,84–86^) and exhibit species specific properties. For example, PDGFRA^+^ cells isolated from rat can be differentiated to Leydig cells, whereas, although the PDGFRA+ isolated cells from the human testes exhibit aspects of MSC characteristics in vitro, they are unable to fully differentiate into Leydig cells, nor can they produce testosterone^41^.

Finally, given the critical role of Tcf21 in the development of other tissues, as well as in aging and disease models^87,88^, we examined commonalities between testis *Tcf21*^+^ cells and similar populations in other organ systems and disease states^69–73^. The *Tcf21*^+^ population in the adult testis molecularly resembles *Tcf21*^+^ fibroblast or fibroblast-like populations that have functional roles in normal tissue maintenance, injury, or disease in other organs such as the heart, lung, and liver. While the response to tissue injury is often context dependent, resulting in fibrosis vs. regeneration, it remains unknown if a single resident mesenchymal population is activated to promote either response depending on the levels of damage or signaling pathways activated or if multiple populations respond and leading to divergent outcomes. Here in response to EDS, the TCF21^lin^ restores Leydig cells but in many cases, fibrosis is often observed in men with impaired spermatogenesis^89–93^. Therefore, whether dysregulation of the TCF21^+^ population may be involved in the pathogenesis of testis fibrosis in certain contexts of infertility remains to be examined. A greater understanding of this population and its regulation may contribute to more informed strategies to restore testis function, as well as the tissue regeneration and tissue repair therapies for other organs^94^.

## Methods

### Lead contact and materials availability

Further information and requests for resources and reagents should be directed to and will be fulfilled by the Lead Contact, Saher Sue Hammoud (hammou@med.umich.edu).

### Mice

All animal experiments were carried out with prior approval of the University of Michigan Institutional Committee on Use and Care of Animals (Animal Protocols: PRO00006047, PRO00008135), in accordance with the guidelines established by the National Research Council Guide for the Care and Use of Laboratory Animals. Mice were housed in the University of Michigan animal facility, in an environment controlled for light (12 h on/off) and temperature (21–23 °C) with ad libitum access to water and food (Lab Diet #5008 for breeding pairs, #5LOD for non-breeding animals).

Colony founders for *Rosa26*^*iDTR*^ (Stock #007900), *Oct4-eGFP* (Stock #004654), *PdgfraDGFRA*^*EGFP*^ (Stock007669), *R26R*^*tdTom*^ mice (Stock #007909), and *Myh11*^*cre-egfp*^ (Stock #007742, maintained on a B6 background) were obtained from Jackson Labs. The *Tcf21*^*mCrem*^ and the *Gli1*^*EGFP*^ were generously provided by Michelle Tallquist and Deb Gumuccio, respectively. EDS injection studies were performed on age-matched C57BL/6 mice obtained from Jackson Labs (Stock #000664) or *Tcf21*^*mCrem*^*:R26*^*RtdTom*^ animals. For detailed mouse strain information, see below. All primers used for genotyping are provided in Supplementary Data 4.

### Interstitial populations single-cell data analysis

#### Single-cell RNA-sequencing analysis comparing somatic cell types in the adult mouse testis

Somatic cells (*N* = 3622) and their cell type classifications were defined by Green et al.^13^. To compare somatic cell populations of the testis we obtained the Euclidean distances for the somatic cell centroids then ordered the cell types using the optimal leaf ordering (OLO) algorithm in R Package Seriation. Based on this analysis, we discovered that the *Tcf21*^+^ population is highly correlated to endothelial, myoid and Leydig cells, but distinct from immune cells and Sertoli cells. To get a better understanding of the functional role of the *Tcf21*^+^ population, we called differentially expressed genes in each somatic cell type using a nonparametric binomial test. The differentially expressed genes have: (1) At least 20% difference in detection rate; (2) a minimum of 2-fold change in average expression levels, and (3) *p* value < 0.01 in the binomial test. Pathway Enrichment analysis for the differentially expressed genes was performed with PANTHER tool v.15 (http://www.pantherdb.org)^95^. Significance of the over- and under-representation of GO Complete Biological Process categories was calculated using Fisher’s exact test and multiple testing correction with the false discovery rate.

#### Ligand–receptor analysis

We used a previously published curated list of ligand–receptor (LR) pairs^34^. We limited the analysis to LR pairs that have either highly variable ligand genes among the 7 somatic cell centroids, or highly variable receptor gene among the 26 germ cell centroids (6 SPG and 20 non-SPG clusters). Thresholds were set for both genes mean and variance across the clusters according to the density. For each ligand–receptor pair we calculated its apparent signaling strength as an “Interaction Score”, defined as the product of the mean expression level of the ligand in one cell type and that of the receptor in another cell type. In all, we calculated such an Interaction Score matrix of cell type pairs for germ (ligand)-soma (receptor) interaction, soma (ligand)-soma (receptor) interaction and soma (ligand)-germ (receptor) interaction (reproduced with permission from^34^, respectively. To extract the general signaling pattern for each interaction matrix, we defined “strong interactions” for each matrix by keeping the highest 5% Interaction Scores for each matrix. We then calculated the number of such strong L–R interactions for each pair of cell types as their overall interaction strength and displayed them as the line width of arrows in the pairwise interaction plots.

#### Cell type correlations across tissues

We downloaded the single-cell counts data from GEO for artery, lung, heart and two liver datasets^69–73^. For the datasets providing cluster information including our testis datasets, we generated expression centroid for each cell type. We then calculated the spearman rank correlation for all cell type pairs between testis and other tissues. For the artery and the liver datasets, the cell type clusters were not provided, so for these datasets we re-analyzed the raw data using Seurat—following the analysis descriptions from the original papers. For parameters that are not specified, we either used default values or set accordingly. To regenerate the clusters for these raw datasets, we used Louvain clustering in Seurat and assigned cell types according to markers listed in the two papers. We then followed the same procedure to calculate cell type expression centroid and spearman rank correlations with cell types from testis. Summary of all correlations are illustrated in the heatmap.

### In vitro differentiation assays

#### Flow cytometry

Testes were collected from adult C57BL/6 (JAX^®^mice, stock #000664) mice and enzymatically and mechanically dissociated into a single-cell suspension. Briefly, testes from adult mice were excised and the tunica albuginea was removed. Seminiferous tubules were transferred to 10 ml of digestion buffer 1 (comprised of Advanced DMEM:F12 media (ThermoFisher Scientific), 200 mg/ml Collagenase IA (Sigma), and 400 units/ml DNase I (Worthington Biochemical Corp)). Tubules were dispersed by gently shaking by hand and a 5-min dissociation at 35 °C/215 rpm. To enrich for interstitial cells, tubules were allowed to settle, and the supernatant was collected, quenched with the addition of fetal bovine serum (FBS) (ThermoFisher Scientific), filtered through a 100 μm strainer and pelleted at 600 g for 5 min. The remaining tubules were then transferred to digestion buffer 2 (200 mg/ml trypsin (ThermoFisher Scientific) and 400 units/ml DNase I (Worthington Biochemical Corp) dissolved in Advanced DMEM:F12 media) and dissociated at 35 °C/215 rpm for 5 min each and quenched with the addition of FBS (ThermoFisher Scientific). The cell pellets from multiple digests were combined and filtered through a 100 μm strainer, washed in phosphate-buffered saline (PBS), pelleted at 600 g for 3 min, and re-suspended in MACS buffer containing 0.5% BSA (Miltenyi Biotec).

The single-cell suspensions were stained with a single antibody or combination of antibodies depending on the experiment. The antibodies used include anti-Ly6a-AlexaFluor 488 (1:100; Biolegend, Cat#108115), Biotinylated anti-Ly6a (1:200; Biolegend, Cat#108103), streptavidin conjugated AlexaFluor 488 (1:1000; Life Technologies Cat# S11223; RRID: AB_2336881), anti-CD73-APC (1:300; Biolegend, Cat#127209), anti-CD90.1-Brilliant Violet 650 (1:300; Biolegend, Cat#202533), anti-CD29-PE/Dazzle 594 (1:300; Biolegend, Cat#102231), anti-CD105-PerCP/Cy5.5 (1:300; Biolegend, Cat#120415), anti-CD45-Brilliant Violet 510 (1:300; Biolegend, Cat#), anti-CD117-PE/Cy7 (c-KIT) (1:300; Biolegend, Cat#105813), and anti-CD34-PE (1:300; Biolegend, Cat#128609).

#### Tri-lineage differentiation assay

Testes were collected from adult C57BL/6 or *Tcf21*^*mCrem*^*:R26R*^*tdTom*^ mice and dissociated into a single-cell suspension and sorted for SCA1^+^/cKITit^−^ or SCA1^+^/TCF21^lin^/cKITit^−^ cells, respectively. For adipogenic differentiation, 3 × 10^4^ cells were plated in a monolayer, cultured for 10 days (Stem ProAdipogenic differentiation kit) and stained with either Oil Red O or Perilipin (1:250, Sigma, Cat#P1873). For chondrogenic differentiation, 5 × 10^4^ cells were plated in micromass, cultured for 21 days (Stem ProChondrogenic differentiation kit) and stained with either Alcian blue or SOX9 (1:250, EMD Millipore, Cat#ABE571). For osteogenic differentiation, 1 × 10^4^ cells were plated in micromass, cultured for 14 days (StemProOsteogenicdifferentiationkit), and stained with either Alizarin red or Osterix (1:250, Abcam, Cat#ab22552)^96^.

#### CFU-assay

Colony-forming unit assays were performed as previously described^97^. Briefly, testes were collected from C57BL/6 or adult *Tcf21*^*mCrem*^*:R26R*^*tdTom*^ males following 3 injections of tamoxifen (2 mg) every other day. Following single-cell dissociations, SCA1^+^/cKITit^−^, SCA1^+^/TCF21^lin^/cKITit^−^, TCF21^lin^/cKITit^−^, or cKIT^+^/SCA1^−^ cells were plated into Corning Primaria 6 well plates at a density of 1000 cells/well. Cells were cultured for 14 days in Mesen Cult MSC medium (StemCell Technologies) and colonies were stained using Giemsa. Colonies were defined as clumps having either >20 or >50 cells.

#### In vitro directed differentiation to Leydig and myoid cells

Testes were collected from adult C57BL/6 or *Tcf21*^*mCrem*^*:R26R*^*tdTom*^ *males*, dissociated into a single-cell suspension, and sorted for SCA1^+^/cKit^−^ cells. Cells were plated into a 24 well, Matrigel-coated plate at a density of 100,000 cells/well in DMEM/F12 supplemented with 10% FBS and 1X Normocin. For myoid cell differentiation, after 18 h media was replaced with differentiation media- DMEM/F12 supplemented with 1X Penicillin/streptomycin, 10 ng/ml PDGFAA, 10 ng/ml PDGFBB, 0.5 µM SAG, 10 ng/ml BMP2, 10 ng/ml BMP4, 10 ng/ml ActivinA, and 1 mM Valproic acid. After the 7-day differentiation protocol, cells were stained for SMA (1:200, Sigma, Cat#A5228). For Leydig cell differentiation, the FACs sorted cells were initially recovered for 18 h in DMEM+10%FBS and then the cells were expanded for 3 days in DMEM/F12 supplemented with 1X Normocin, 10 ng/ml PDGFAA, 10 ng/ml PDGFBB, 0.5 µM SAG, and 10 ng/ml FGF2. After 3 days, the expansion media was replaced with differentiation media- DMEM/F12 supplemented with 1X Penicillin/streptomycin, 10 ng/ml PDGFAA, 10 ng/ml PDGFBB, 0.5 µM SAG, 10 ng/ml FGF2, 5 mM LiCl_2_, and 10 µM DAPT. After 10 days in differentiation media, cells were stained for SF1 (1:100, CosmoBio, Cat#KAL-KO610). Media was collected every other day for testosterone measurements.

### Clonal expansion and directed differentiation of TCF21^lin^ cells

To assess multipotency of TCF21^lin^ cells, testes were collected from adult *Tcf21*^*mCrem*^*:R26R*^*tdTom*^ males and dissociated into a single-cell suspension. Individual TCF21^lin^/cKit^−^, SCA1^+^/TCF21^lin^/cKit^−^, SCA1^+^/TCF21^lin^/cKit^−^/Cd105^+^ or SCA1^+^/TCF21^lin^/cKit^−^/Cd105^−^ cells were sorted into Corning Primaria 96-well plates and cultured in Mesen Cult mouse MSC media for ~3 weeks to allow for colony formation. Individual colonies were then directed to differentiate to either a myoid or Leydig cell fate following the directed differentiation protocols described above. Cells were then stained for either SMA (for myoid cells, 1:200, Sigma, Cat#A5228) or SF1 (Leydig cells, 1:100, CosmoBio, Cat#KAL-KO610).

#### Drop-seq analysis of the Leydig cell differentiation time-course analysis

Drop-seq was performed on cells collected from various points of differentiation, where a single sample per timepoint was diluted to 280 cells/µl and processed as described previously^98^. Briefly, cells, barcoded microparticle beads (MACOSKO-2011-10, Lots 113015B and 090316, ChemGenes Corporation), and lysis buffer were co-flown into a microfluidic device and captured in nanoliter-sized droplets. After droplet collection and breakage, the beads were washed, and cDNA synthesis occurred on the bead using Maxima H-minus RT (ThermoFisher Scientific) and the Template Switch Oligo. Excess oligos were removed by exonuclease I digestion. cDNA amplification was done for 15 cycles from pools of 2000 beads using Hot Start Ready Mix (Kapa Biosystems) and the SMART PCR primer. Individual PCRs were purified and pooled for library generation. A total of 600 pg of amplified cDNA was used for a NexteraXT library preparation (Illumina) with the New-P5-SMARTPCR hybrid oligo, and a modified P7 Nexteraoligo with 10 bp barcodes. Sequencing was performed on a NovaSeq (Illumina) for read 2 length of 94 nt with the Read1 Custom Seq primer. Oligosequences are the same as previously described^13,98^.

#### Single-cell RNA-seq analysis across time points

The paired-end Drop-seq data from days 4, 7, and 14 of in vitro Leydig cell differentiation were sequenced in the same batch and were processed using *Drop-seq tools* v1.13 from the McCarroll laboratory as previously described^98,99^. Specifically, the reads were aligned to the mouse reference genome (GRCm38, version 38) using *STAR* v2.7.1a^100^. The pipeline generated digital gene expression matrices with genes as rows and cells as columns that served as the starting point for downstream analyses.

The cells from each time point were first filtered by cell size and integrity—cells with <500 detected genes or with >10% of transcripts corresponding to mitochondria-encoded genes were removed, resulting in 973–2980 pass-QC cells for the three time points, for a total of 6124 cells. Among the retained cells, the average number of detected genes per cell was ~1888, and the average number of UMIs was ~4838. For each cell, we normalized transcript counts by (1) dividing by the total number of UMIs per cell and (2) multiplying by 10,000 to obtain a transcripts-per-10K measure, and then log-transformed it by E = ln(transcripts-per-10K+1).

For each time point, we standardized the expression level of each gene across cells by using (E-mean(E))/sd(E) and performed PCA using highly variable genes (HVG). We obtained 4 clusters using Louvain-Jaccard clustering with top PCs by R package *Seurat* (v2.3.4). We calculated cluster centroids and ordered the clusters by minimizing pairwise Euclidean distance of cluster centroids in R package *Seriation*. We evaluated batch effect by comparing the top PC placements and rank correlation of ordered cluster centroids across the 3 time points. Differentially expressed markers for each cluster were obtained by comparing it against all other clusters using a nonparametric binomial test, requiring at least 20% higher detection rate, a minimum of 1.5-fold higher average expression level, and *p* value < 0.01. Clusters were defined based on known markers.

We extracted the somatic cells from the INT4 dataset from^13^. This dataset was enriched for the *Tcf21*^*+*^ interstitial population and was used as the starting time point: day 0. We merged the 3 datasets of in vitro Leydig differentiation with the somatic cells of INT4, for 6619 good-quality cells and 24,698 detected genes. These cells on average have 1837 detected genes and 4623 UMIs per cell. We selected 2344 HVG genes in the merged dataset and did PCA using HVG. We performed t-SNE, UMAP and Louvain-Jaccard clustering using top PCs. We obtained 10 clusters initially, and ordered the clusters as described above. Based on differentially expressed markers for each cluster and rank correlation across the cluster centroids, we decided to merge 3 clusters (clusters 4–6) and identified them as ECM myofibroblast. We merged 2 other clusters (clusters 7–8) as differentiating myofibroblast. This led to the identification of 7 cell types for in vitro Leydig differentiation—(1) Endothelial, (2) Interstitial progenitor, (3) Proliferating progenitor, (4) ECM myofibroblasts, (5) Differentiating myofibroblasts, (6) Differentiating Leydig, and (7) Leydig. We did pseudotemporal ordering of cells from the 4 time points (days 0, 4, 7, and 14) by *Monocle3* and visualized the single-cell trajectory in UMAP space.

We compared our in vitro Leydig differentiation data with those of fetal mouse gonads and adult mouse testis somatic cells. Specifically, we calculated the rank correlation between our 7 cell type centroids of Leydig differentiation data with the 6 cell type centroids from NR5A1-eGFP^+^ progenitor cells from E10.5 to E16.5 fetal male mouse gonads^33^ using markers present in both data (*N* = 2692). We also calculated the rank correlation between our 7 cell type centroids with the 7 cell type centroids from adult mouse testis (Green et al.^13^) using the union of markers present in both datasets (*N* = 1769).

### TCF21 lineage tracing

#### TCF21 lineage tracing analysis and colocalization with immunofluorescence in fetal and adult testis and ovary

*Tcf21*^*mCrem*^*:R26R*^*tdTom*^ or *Tcf21*^*mCrem*^*:R26R*^*tdTom*^; *Oct4-eGFP* timed-pregnant females were administrated with a single dose of 1 mg tamoxifen via gavage at E9.5, E10.5, E11.5, or E12.5. Embryos were obtained at E11.5, E17.5, or E19.5 via C-section. Tail clippings from the embryos were used to identify sex and genotype. Embryonic gonads were fixed in 4% paraformaldehyde (PFA) at 4 °C for 1 h, transferred to 30% sucrose in 1xPBS at 4 °C overnight and embedded in OCT (Surgipath cryo-gel, Leica #39475237). To analyze the TCF21^lin^ contribution in adult testis and ovaries, the E19.5 pups obtained by C-section were fostered to CD1 females. The foster mice were euthanized at 10 weeks. Adult testes and ovaries were fixed in 4% PFA at 4 °C overnight, transferred to 30% sucrose in 1xPBS at 4 °C for overnight and embedded in OCT.

For immunofluorescence, 7–10 micron thick OCT sections were cut using a Leica CM3050S cryostat and sections were refixed with 4% PFA for 10 min and permeabilized by incubation in 0.1% Triton in PBS for 15 min. Sections were blocked in 1xPBS supplemented with 3% BSA and 500 mM glycine for 1 h at room temperature and co-stained with FGF5, SOX9, 3β-HSD, CD34, SMA, VASA, COUPTFII, CD31, SF1, WT1, FOXL2, and DsRed antibodies. The primary antibodies and concentrations used are summarized in Supplementary Data 5. All secondary antibodies (Alexa-488-, Alexa-568-, and Alexa-647-conjugated secondary antibodies; Life Technologies/MolecularProbes) were all used at a 1:1000 dilution. DAPI was used as a nuclear counterstain at 1:1000. Representative images were taken with a ZeissAX10 epifluorescence microscope, a Leica SP8 confocal microscope, or a Nikon A1R-HD25 confocal microscope and processed with ImageJ.

#### Quantification of immunofluorescence colocalization

Tissue sections were stained for immunofluorescence as described above and >20 images per testis were imaged with a ×40 1.2NA objective on a ZeissAX10 epifluorescence microscope, all at a single z-section. The percent overlap between the TCF21^lin^ population and several marker proteins was done using accustom written ImageJ macro (available upon request). Briefly, nuclear regions of interest (ROIs) were created from DAPI staining by blurring with a Gaussian filter, making the image binary, separating overlapping nuclei with a watershed function, then saving the outline of each binary nucleus to ImageJ’s ROI manager. The signal from TCF21^lin^ and the immunostaining were made binary using ImageJ’s automatic thresholding function and the overlap of the binary stain and the nuclear ROI was measured using Image J’s Measurement function. Cells positive for each stain and double-positive cells were sorted and identified in Microsoft Excel. The quantification from each testis was the sum of all quantified images taken from a single testis. The macro was optimized by contrasting its results to manual quantification from at least five images per immunostaining.

#### Lineage-tracing analysis of the Tcf21^+^ population in the aged testis

Five or eight-week-old *Tcf21*^*mCrem*^*:R26R*^*tdTom*^ males were injected with a single dose of 2 mg tamoxifen intraperitoneally. The testes were collected 1 week (as control) or 1-year past injection. The testes were fixed in 4% PFA at 4 °C overnight, transferred to 30% sucrose in 1xPBS at 4 °C overnight and embedded in OCT. The sections were co-stained for SF1 (1:100, CosmoBio, Cat#KAL-KO610). Overlap of SF1 and tdTomato were counted manually per field; ~25–100 fields per biological replicate per condition were collected.

### In vivo ablation of Leydig cells

#### Ethane dimethane sulfonate (EDS) injections

EDS (AABlocks, Cat. No: 4672-49-5) was dissolved in DMSO at a concentration of 150 mg/mL. Working solutions of EDS were further diluted in PBS to a final concentration of 50 mg/mL. A total of 300 mg/kg of EDS was injected intraperitoneally every other day for 2 days into C57BL/6 age-matched or *Tcf21*^*mCrem:R26RtdTom*^ mice. Testes and sera were collected 12 h, 24 h, 4 days, 7 days, 14 days, or 21 days past final injections. Negative controls were given vehicle treatment of DMSO: PBS without EDS.

#### Diphtheria toxin Injections

DTX (Sigma) was diluted in PBS at a concentration of 2 mg/ml. A final concentration of 150 ng DTX was injected intraperitoneally every day for 3 or 4 days into *Myh11*^*cre-egfp*^*;Rosa26*^*DTR/+*^ male mice. Testes were collected 12 h, and 4 days past final injections. Littermates that are Myh11-Cre-recombinase negative were given DTX and served as controls.

#### TUNEL staining

Testes were collected, fixed in 4% PFA for ~16 h at 4 °C, dehydrated in ethanol wash series, and embedded in paraffin. Five-micron FFPE tissue sections were deparaffinized, rehydrated, and permeabilized in 20 μg/ml Proteinase K solution for 15 min at room temperature. Samples were further processed following the Promega Dead End Colorimetric TUNEL kit according to the manufacturer instructions. All images collected used a Leica Leitz DMRD microscope.

#### Hormone measurements

Testosterone measurements were performed by the University of Virginia Center for Research in Reproduction Ligand Assay and Analysis Core.

#### FFPE immunofluorescence

Whole testes were fixed in 4% PFA overnight at 4 °C and processed for formalin fixed paraffin embedding as described in (Fisher et al. 2008). Five-micron FFPE tissue sections were deparaffinized by incubation in Histoclear 3× for 5 min, followed by incubation in 100% EtOH 2× for 5 min, 95% EtOH 2× for 5 min, 80% EtOH 1× for 5 min, 70% EtOH 1× for 5 min, 50% EtOH 1× for 5 min, 30% EtOH 1× for 5 min, and deionized water 2× for 3 min each. Tissue sections were permeabilized by incubation in 0.1% Triton in PBS for 15 min. For all antibodies, antigen retrieval was performed by boiling in 10 mM sodium citrate, pH 6.0 for 30 min. Sections were blocked in 1xPBS supplemented with 3% BSA and 500 mM glycine for 3 h at room temperature. Endogenous peroxidases and alkaline phosphatases were blocked by a 10-min incubation in BloxAll solution (VectorLabs, Cat. No: SP-6000). The primary antibodies and concentrations used are listed below. Alexa-488-, Alexa- 555-, and Alexa-647-conjugated secondary antibodies (Life Technologies/MolecularProbes) were all used at 1:1000. DAPI was used as a nuclear counterstain. For quantification, the overlap of SF1 and tdTomato were counted manually per field; ~25–50 fields per biological replicate per condition were collected.

#### Cell diameter measurements

For all cell diameter measurements, FFPE slides were stained as described above and co-stained with SF1 to mark Leydig cells (1:100, CosmoBio, Cat#KAL-KO610) and 488-wheat germ agglutinin (WGA, Biotium Cat. No: 29022-1) to mark cell perimeter. A tubule was centered in the field of view at ×40 magnification and an image was taken. At least 70 well-defined Leydig cells, marked by both SF1 and clear visible cell perimeter by WGA, were counted per condition. Cell diameter was measured manually using ImageJ’s Line and Measure functions.

#### FFPE immunohistochemistry

Whole testes were fixed in 4% PFA overnight at 4 °C, processed and deparaffinized as described above. The primary antibodies and concentrations used are listed in Supplementary Data 5. Horseradish peroxidase and alkaline phosphatase conjugated secondary antibodies (Abcam) were all used at a 1:100 concentration and left to incubate for 1-h at room temperature. Slides were rinsed of developing solution under running DI water for 1 min and mounted in per mount.

#### Transplantation of TCF21^lin^ cells into the testis of WT EDS treated or *Myh11*^*cre-egfp*^*;Rosa26*^*DTR/+*^ animals

6–18 weeks *Tcf21*^*mCrem*^*:R26R*^*tdTom*^ male mice were injected with 1 mg tamoxifen intraperitoneally every other day for a total of three injections. Testes were dissociated into a single-cell suspension and the SCA1^+^/cKITit^−^ stained cells were collected by FACs, as described above. For each animal ~65,000–150,000 cells were diluted in 10 μl of MEM media plus Trypan Blue and were injected into the interstitium via the rete testes of either EDS-treated C57BL/6 animals 24 hpfi of EDS or into *Myh11*^*cre-egfp*^*;Rosa26*^*DTR/+*^ mice 24 hpfi of DTX. As a control, 10 μL of MEM media plus Trypan Blue was injected into the contralateral testis of each experimental animal. Testes were collected at 24 h post transplant (hpt), 4 days post transplant (dpt) and 7 dpt for FPPE processing as described above.

### Reporting summary

Further information on research design is available in the Nature Research Reporting Summary linked to this article.

## Supplementary information

Supplementary Information

Description of Additional Supplementary Files

Supplementary Dataset1

Supplementary Dataset2

Supplementary Dataset3

Supplementary Dataset4

Supplementary Data 5

Reporting Summary

## Data Availability

Single-cell RNA-seq data files are available in “GSE151337”. All other relevant data supporting the key findings of this study are available within the article and its Supplementary Information files or from the corresponding author upon reasonable request. A reporting summary for this article is available as a Supplementary Information file. Source data are provided with this paper.

## Source data

Source Data

## Code availability

Codes used in this analysis were deposited onto GitHub: 10.5281/zenodo.4743036.
