# Supplementary material for: TCF21+ mesenchymal cells contribute to testis somatic cell development, homeostasis, and regeneration in mice
Source: Nat Commun. 2021 Jun 23;12:3876. doi: 10.1038/s41467-021-24130-8 (PMC8222243; doi:10.1038/s41467-021-24130-8)
Supplement: Supplementary file 1 — Supplementary Information [file 41467_2021_24130_MOESM1_ESM.pdf]

Supplementary Figures

Tcf21+ mesenchymal cells contribute to testis somatic cell development, homeostasis, and regeneration

Shen et al

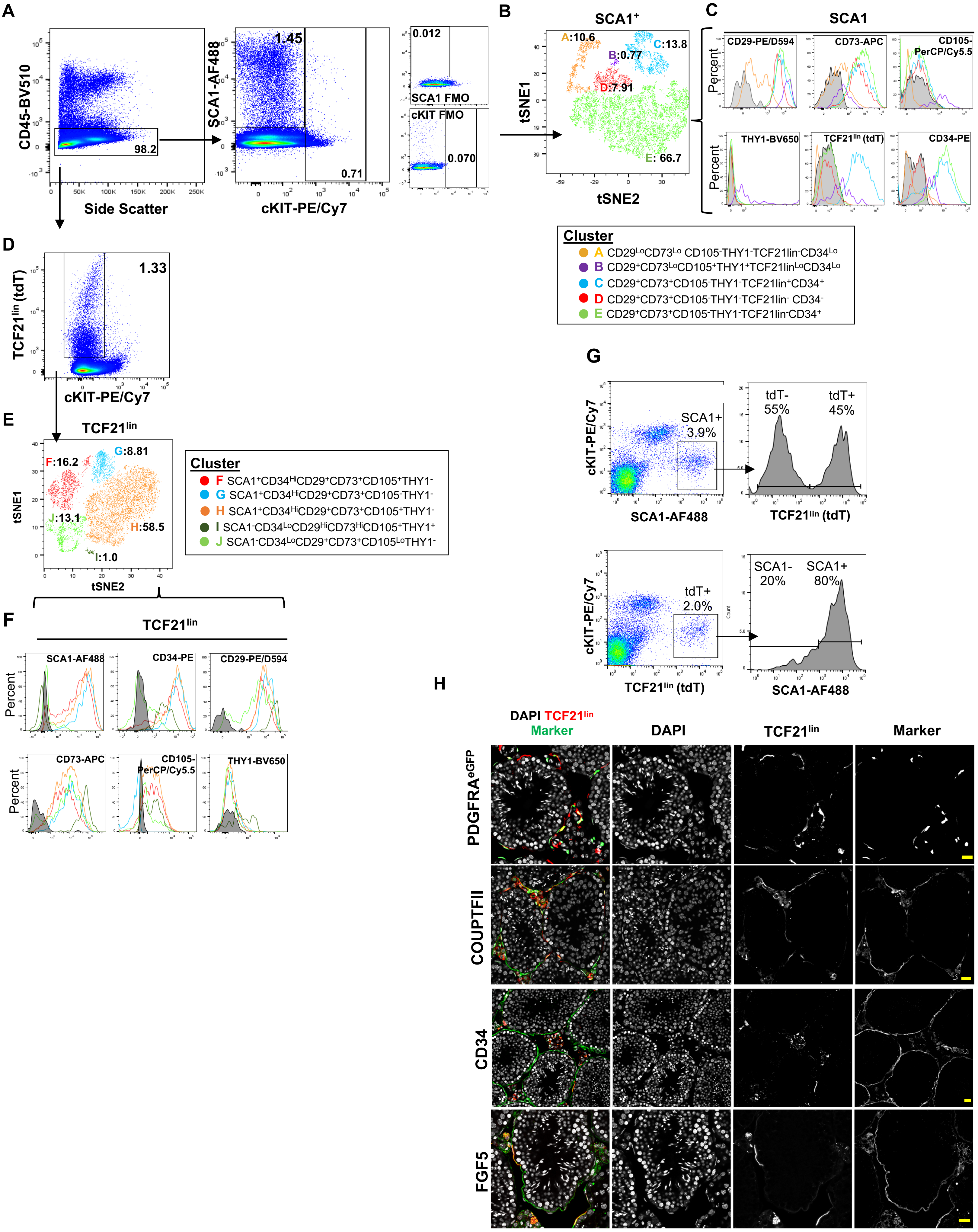
A
CD45-BV510
Side Scatter
98.2
SCA1-AF488
cKIT-PE/Cy7
1.45
0.71
0.012
SCA1 FMO
cKIT FMO
0.070
B
SCA1+
A:10.6
B:0.77
C:13.8
D:7.91
E: 66.7
tSNE1
tSNE2
C
SCA1
CD29-PE/D594
CD73-APC
CD105-PerCP/Cy5.5
THY1-BV650
TCF21lin (tdT)
CD34-PE
Percent
Cluster
A CD29LoCD73Lo CD105-THY1-TCF21lin-CD34Lo
B CD29+CD73LoCD105+THY1+TCF21linLoCD34Lo
C CD29+CD73+CD105-THY1-TCF21lin+CD34+
D CD29+CD73+CD105-THY1-TCF21lin- CD34-
E CD29+CD73+CD105-THY1-TCF21lin-CD34+
D
TCF21lin (tdT)
cKIT-PE/Cy7
1.33
E
TCF21lin
F:16.2
G:8.81
H:58.5
J:13.1
I:1.0
tSNE1
tSNE2
Cluster
F SCA1+CD34HiCD29+CD73+CD105+THY1-
G SCA1+CD34HiCD29+CD73+CD105-THY1-
H SCA1+CD34HiCD29+CD73+CD105+THY1-
I SCA1-CD34LoCD29HiCD73HiCD105+THY1+
J SCA1-CD34LoCD29+CD73+CD105LoTHY1-
F
TCF21lin
SCA1-AF488
CD34-PE
CD29-PE/D594
CD73-APC
CD105-PerCP/Cy5.5
THY1-BV650
Percent
G
cKIT-PE/Cy7
SCA1+
3.9%
SCA1-AF488
tdT-
55%
tdT+
45%
TCF21lin (tdT)
tdT+
2.0%
SCA1-
20%
SCA1+
80%
Count
H
DAPI TCF21lin
Marker
DAPI
TCF21lin
Marker
PDGFRAeGFP
COUPTFII
CD34
FGF5

Supplementary Figure 1: The SCA1$^+$ and Tcf21^{Lin} populations are molecularly heterogeneous and expresses multiple mesenchymal progenitor markers. (A) Gating strategy used to identify interstitial SCA1$^+$ cells in the testis. The first plot shows side scatter area and CD45 fluorescent intensity used to eliminate immune cell populations (selection of SCA1$^+$**,** CD45$^-$). The second plot is a SCA1$^+$ and cKIT$^+$ fluorescent intensity plot used to remove Leydig cells (selection of SCA1$^+$**,** CD45$^-$**,** cKIT$^-$). All positive gates were determined using FMO isotype controls. **(B)** t-SNE analysis (perplexity 35,300 iterations) of thousands of cells co-stained for TCF21^{lin}, CD73, CD105, Thy1, CD29, and CD34 identifies five SCA1$^+$ subtypes (Clusters A-E) based on the signal intensity of various markers. **(C)** Fluorescent intensity histograms of the 6 markers for the 5 clusters defined in panel B, depicted with corresponding colors. Isotype controls for each marker are indicated with a grey shaded peaks. **(D)** Gating strategy used to characterize interstitial Tcf21^{lin} cells in the testis, similar to A. Note no FMO isotype control shown for Tcf21 due to genetic labeling strategy. **(E)** t-SNE analysis (perplexity 35,300 iterations) of thousands of cells co-stained for SCA1, CD73, CD105, THY1, CD29, and CD34 identifies five Tcf21$^{\mathbf{lin}}$ clusters (Clusters F-J) based on the signal intensity of various markers. **(F)** Fluorescent intensity histogram for each of 6 markers across the 5 clusters defined in panel E, depicted with corresponding colors. Isotype controls for each marker are indicated with grey shaded peaks. Data was collected from a pool of 2-3 animals. **(G)** Characterizing the overlap between the Tcf21^{lin} (Tdtomato) and SCA1. Data collected from a pool of 2-3 animals. **(H)** Immunofluorescence overlay of Tcf21^{lin} with PDGFRA^{eGFP}(n=1), COUPTFII, CD34**,** and FGF5 (n=2) expression in the adult mouse testis. Scale bars: 20μm. All images were taken with the same magnification.

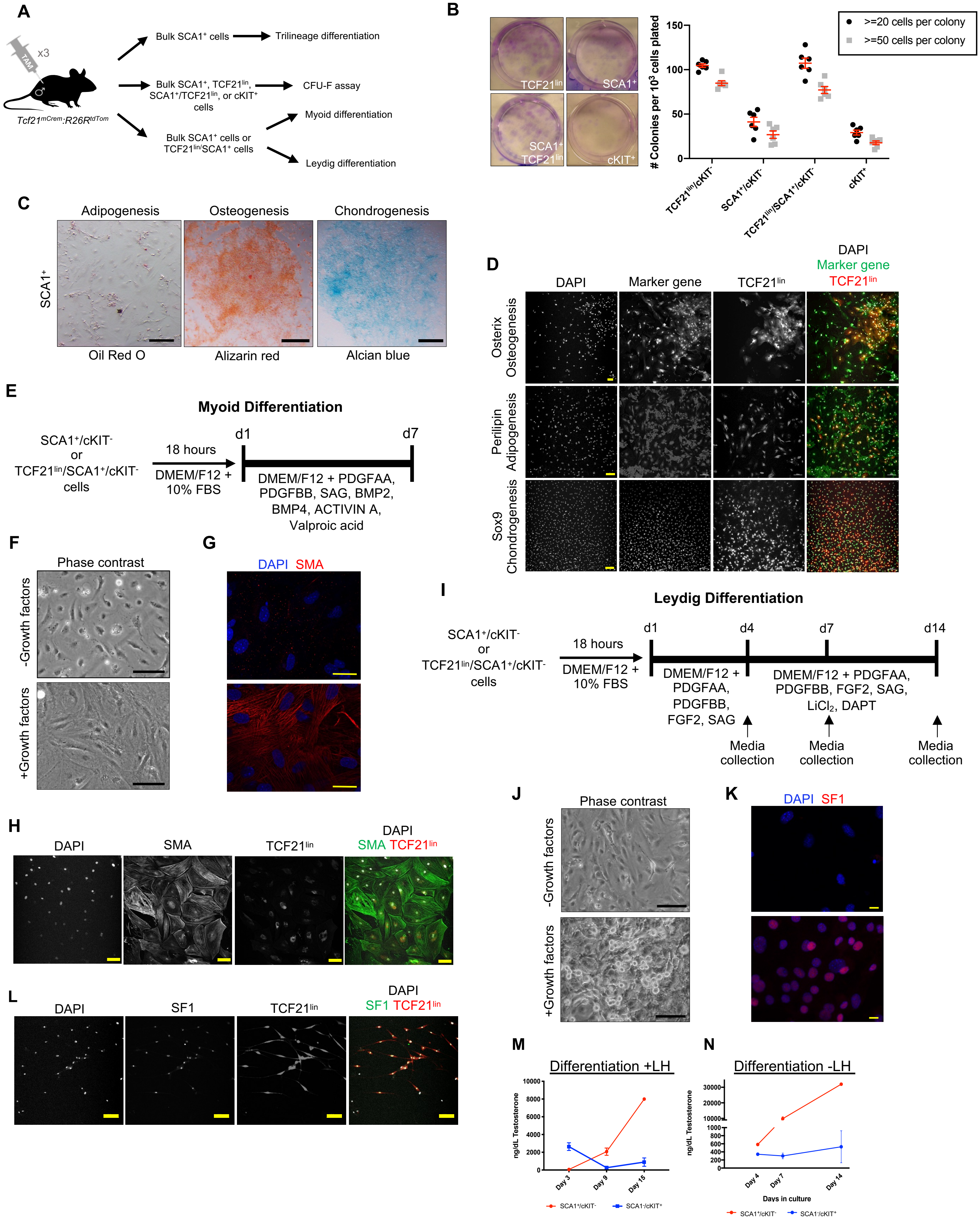
A
x3
TAM
Tcf21mCrem:R26RtdTom
Bulk SCA1+ cells
Trilineage differentiation
Bulk SCA1+, TCF21lin, SCA1+/TCF21lin, or cKIT+ cells
CFU-F assay
Bulk SCA1+ cells or TCF21lin/SCA1+ cells
Myoid differentiation
Leydig differentiation
B
TCF21lin
SCA1+
SCA1+ TCF21lin
cKIT+
Colonies per 10³ cells plated
>=20 cells per colony
>=50 cells per colony
TCF21lin/cKIT-
SCA1+/cKIT-
TCF21lin/SCA1+/cKIT-
cKIT+
C
Adipogenesis
Osteogenesis
Chondrogenesis
SCA1+
Oil Red O
Alizarin red
Alcian blue
D
DAPI
Marker gene
TCF21lin
DAPI
Marker gene
TCF21lin
Osterix Osteogenesis
Perilipin Adipogenesis
Sox9 Chondrogenesis
E
Myoid Differentiation
SCA1+/cKIT-
or
TCF21lin/SCA1+/cKIT-
cells
18 hours
DMEM/F12 + 10% FBS
d1
d7
DMEM/F12 + PDGFAA, PDGFBB, SAG, BMP2, BMP4, ACTIVIN A, Valproic acid
F
Phase contrast
-Growth factors
+Growth factors
G
DAPI SMA
H
DAPI
SMA
TCF21lin
DAPI
SMA TCF21lin
I
Leydig Differentiation
SCA1+/cKIT-
or
TCF21lin/SCA1+/cKIT-
cells
18 hours
DMEM/F12 + 10% FBS
d1
d4
d7
d14
DMEM/F12 + PDGFAA, PDGFBB, FGF2, SAG
DMEM/F12 + PDGFAA, PDGFBB, FGF2, SAG, LiCl2, DAPT
Media collection
Media collection
Media collection
J
Phase contrast
-Growth factors
+Growth factors
K
DAPI SF1
L
DAPI
SF1
TCF21lin
DAPI
SF1 TCF21lin
M
Differentiation +LH
ng/dL Testosterone
Day 3
Day 9
Day 15
SCA1+/cKIT-
SCA1-/cKIT+
N
Differentiation -LH
ng/dL Testosterone
Day 4
Day 7
Day 14
Days in culture
SCA1+/cKIT-
SCA1-/cKIT+

Supplementary Figure 2: The Tcf21^{lin} population is a mesenchymal progenitor that can be differentiated to Leydig or myoid cells. (A) Schematic representation for trilineage differentiation, CFU-F, and myoid/Leydig cell differentiation. **(B)** Enrichment of clonogenic cells in the Tcf21^{lin} population. Left panels are CFU-F representative images and right panel is quantification of the number of colonies with either >20 or >50 cells (n=6 wells). Data are presented as mean ± SEM. **(C)** Representative images of SCA1^{+} trilineage differentiation. Adipogenic differentiation validated using Oil Red O, chondrogenic differentiation using Alcian blue, and osteogenic differentiation using Alizarin red. Representative images from n=4 biological replicas each with n=3 technical replicas. Scale bar: 200 μm for adipogenesis; 1 mm for osteogenesis and chondrogenesis. **(D)** Representative images from SCA1^{+}/Tcf21^{lin} trilineage differentiation: Anti-Perilipin, anti-SOX9, and anti-Osterix staining is used to confirm the generation of Adipocytes, Chondrocytes, and Osteocytes respectively (n=4 wells per condition). Scale bar: 100 μm for each row. All images in a series taken with the same magnification. **(E)** Myoid differentiation schema for bulk SCA1^{+}/cKIT^{-} or SCA1^{+}/TCF21^{lin}/cKIT^{-} populations. **(F)** Monitoring morphological changes of SCA1^{+}/cKIT^{-} cells in the presence or absence of myoid differentiation cocktail via phase contrast **(**Scale bar:100 μm), **(G)** SMA expression in terminally differentiated Myoid cells with and without growth factors **(**Scale bar: 25 μm). The representative images are from n= 8 biological replicates and n=3 independent experiments. **(H)** Representative images of SMA expression in SCA1^{+}/TCF21^{lin}/cKIT^{-} differentiated cells. Scale bar: 100 μm. A total of n=3 independent experiments with n=8 technical replicates were performed. **(I)** Leydig cell differentiation scheme for bulk SCA1+/cKIT-or SCA1+/TCF21lin/cKIT-populations. Cell morphology in the presence or absence of a differentiation media via phase contrast (Scale bar:100 μm, **J**) and expression of SF1 in SCA1+ cells **(K)** or SCA1+/TCF21lin/cKIT-cells (Scale bar:10 μm, **L**) after 14 days of culture. Scale bar: 100 μm. A total of n=3 independent experiments with n=8 technical replicates for **J-L**. **(M)** Media testosterone levels from SCA1+/cKIT- and cKIT+/SCA1-cells at 3 (n=6 per condition), 9, and 15 days of culture (n=4 per condition) in the presence of LH. Data are presented as mean ±SEM. **(N)** Media testosterone levels measured from SCA1+/cKIT-and cKIT+/SCA1-cells at 4, 7, and 14 days (n=3 per condition) of culture in the absence of LH. Data are presented as mean ±SEM.

A

Stevant *et al.*, 2018
Somatic cell clusters of the fetal mouse testis

In vitro differentiation Leydig cell clusters

	C1.Endothelial	C2.EarlyProg	C3.IntProg	C4.Pre.Sertoli	C5.FetalLeydig	C6.Sertoli
1.Endothelial	0.49	0.35	0.39	0.35	0.35	0.36
2.IntProg	0.42	0.41	0.56	0.40	0.46	0.39
3.ProlifProg	0.46	0.54	0.64	0.39	0.48	0.45
4.ECMmyofibroblast	0.46	0.49	0.58	0.43	0.45	0.41
5.DiffMyofibroblast	0.46	0.49	0.60	0.41	0.45	0.40
6.DiffLeydig	0.43	0.46	0.61	0.45	0.53	0.46
7.Leydig	0.39	0.42	0.55	0.49	0.58	0.50

B

Green *et al.*, 2018
Somatic cell clusters of the adult mouse testis

In vitro differentiation Leydig cell clusters

	InnateLymphoid	Macrophage	Endothelial	Myoid	*Tcf21*+Int	Leydig	Sertoli
1.Endothelial	0.26	0.36	0.89	0.59	0.54	0.43	0.35
2.IntProg	0.32	0.61	0.58	0.63	0.98	0.73	0.41
3.ProlifProg	0.39	0.50	0.56	0.63	0.74	0.58	0.37
4.ECMmyofibroblast	0.33	0.50	0.56	0.66	0.77	0.62	0.41
5.DiffMyofibroblast	0.37	0.53	0.58	0.67	0.80	0.61	0.36
6.DiffLeydig	0.28	0.54	0.51	0.61	0.85	0.73	0.42
7.Leydig	0.24	0.47	0.44	0.53	0.76	0.84	0.47

Supplementary Figure 3: *In vitro* derived Leydig cells resemble adult Leydig cells present in vivo. (A) Rank correlation of the seven centroids from the *in vitro* Leydig time-course differentiation with 6 cluster centroids derived from previously published Nr5a1^{+}GFP^{+} progenitor cells from E10.5 – E16.5 male mouse gonads[33], and **(B)** previously published adult mouse somatic cells[13].

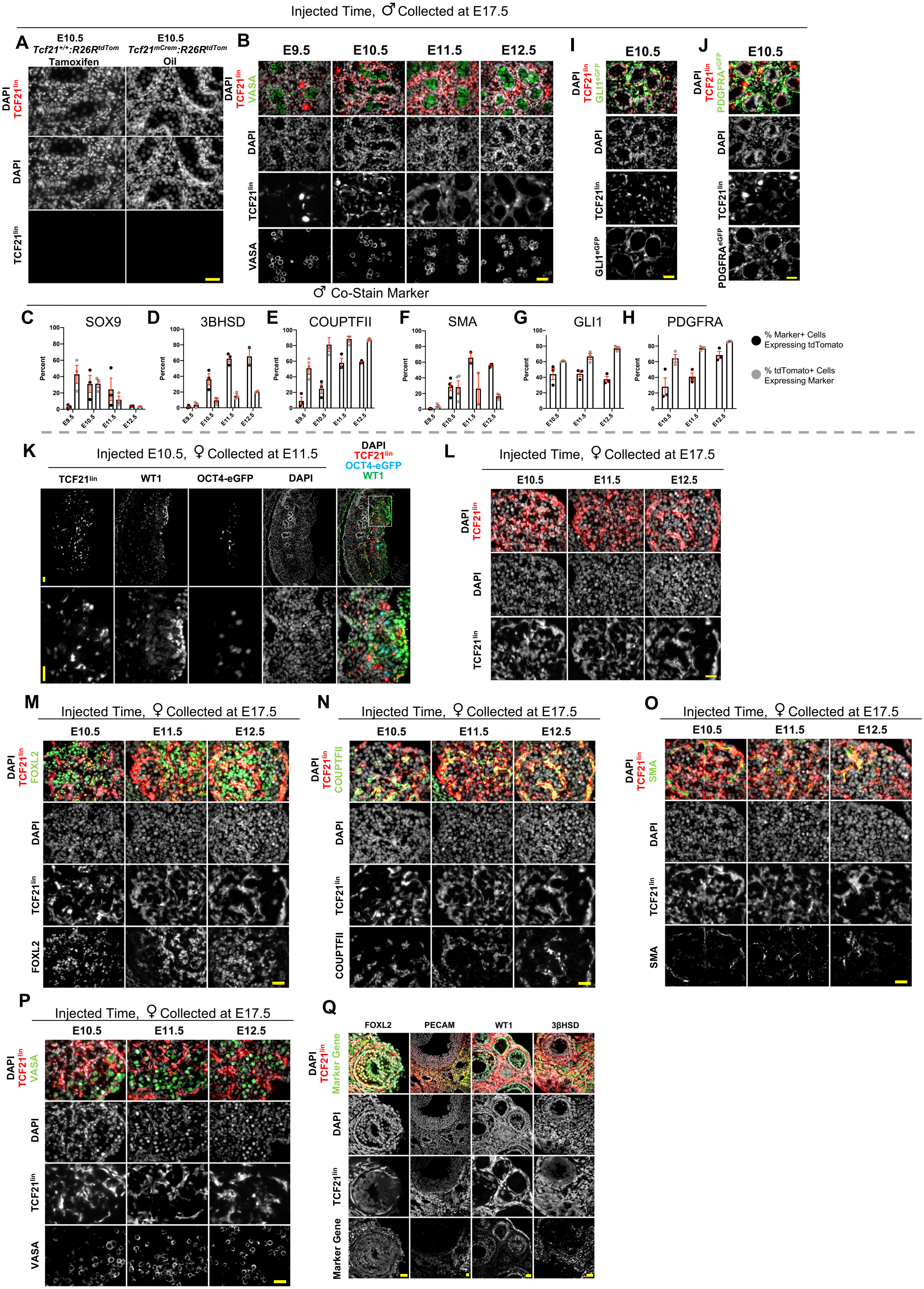

Injected Time, ♂ Collected at E17.5
A
E10.5 Tcf21+/+:R26RtdTom Tamoxifen
E10.5 Tcf21mCrem:R26RtdTom Oil
DAPI TCF21lin
DAPI
TCF21lin
B
E9.5
E10.5
E11.5
E12.5
DAPI TCF21lin VASA
VASA
I
E10.5
DAPI TCF21lin GLI1eGFP
GLI1eGFP
J
PDGFRAeGFP
♂ Co-Stain Marker
C SOX9
D 3BHSD
E COUPTFII
F SMA
G GLI1
H PDGFRA
Percent
% Marker+ Cells Expressing tdTomato
% tdTomato+ Cells Expressing Marker
K
Injected E10.5, ♀ Collected at E11.5
TCF21lin
WT1
OCT4-eGFP
DAPI
L
Injected Time, ♀ Collected at E17.5
M
FOXL2
N
COUPTFII
O
SMA
P
Q
PECAM
3βHSD
Marker Gene

Supplementary Figure 4: The fetal Tcf21^{lin} population is a bipotential somatic progenitor. (A) *Tcf21$^{+/+}$:R26R^{tdTom}* or *Tcf21^{mCrem}:R26R^{tdTom}* time pregnant females (E10.5) were treated with Tamoxifen (left panels, n=3) or corn oil (right panels, n=3) , respectively, confirming specificity and tightness of Cre expression. (**B)** Co-immunostaining of the Tcf21^{lin} with germ cell marker Vasa **(Ddx4; green)** in the male fetal testis (collected at different injection timepoints, n=2 for E9.5, n=3 for E10.5, n=1 for E11.5, n=1 for E12.5). **(C-H)** Quantification of the percentage of various testis somatic cells co-labeled with Tcf21^{lin} (black bars) and percentage of cells in the Tcf21^{lin} population co-expressing testis somatic markers (gray bars) at different embryonic days (n=2-4 per timepoint per marker). Data are presented as mean ±SEM. **(I, J)** Colocalization of Tcf21^{lin} and Gli1-egfp **(I, n=6)**, PDGFRA^{eGFP} **(J, n=7)**, and the nuclear counterstain DAPI. **(K)** Tcf21^{lin} cells in the E11.5 fetal ovary are present in the coelomic epithelium and mesonephros (n= 1 whole ovary). Overlaying Tcf21^{lin} (Tdtom^{+}) cells with WT1^{+} cells **(green; (L)** E10.5-E12.5 Tcf21^{lin} cells label somatic cells broadly in the fetal ovary at E17.5 (n=12 for E10.5, n=7 for E11.5, n=6 for E12.5). **(M-P**) Overlay of the Tcf21^{lin} with granulosa cell marker FOXL2 **(green; M,** n=2-3 per timepoint**)**, the interstitial cell marker COUPTFII (**Nr2F2, green; N,** n=2-3 per timepoint); the smooth muscle marker SMA **(green; O,** n=2-4 per timepoint**)**; the germ cell marker VASA **(DDX4, green; P. n**=2-3 per timepoint**)**. **(Q)** The E10.5 fetal Tcf21^{lin} contributes to multiple somatic cell types in the adult ovary (n=3-4 per marker). In all panels the nuclear counterstain is DAPI. Scale bars: 20 µm for all panel except **K** and **Q. K**: 20 µm for each row, **Q**: 20 µm for each column. All images within a panel were taken at the same magnification.

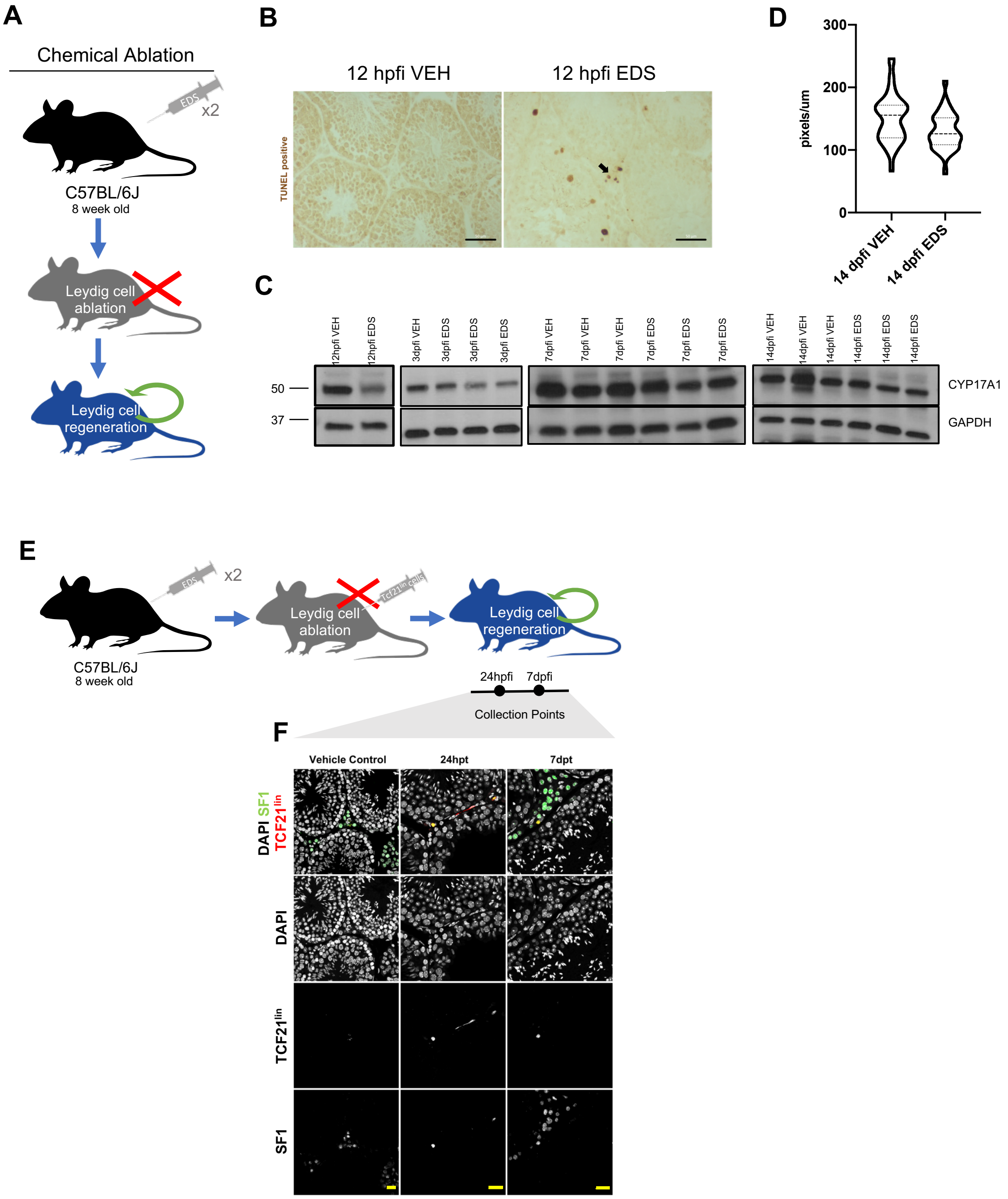

Supplementary Figure 5: Ethane Dimethane Sulfonate (EDS) treatment ablates Leydig cells in the adult testis.
(A) Schematic representation of the chemical method used to ablate Leydig cells. **(B)** Representative images of apoptotic Leydig cells by TUNEL staining in adult C57BL/6 testes collected 12hpfi of EDS (vehicle n=3, EDS n=4). Scale bars: 50 μm for both panels. **(C)** Western blot illustrating Cyp17a1 protein expression levels at 12hpfi, 3dpfi, 7dpfi, and 14dpfi in C57BL/6 animals after EDS or vehicle (VEH) treatment (representative of n=3-4 per condition per timepoint). **(D)** Average Leydig cell diameter measurements 14dpfi of EDS or vehicle in C57BL/6 animals, after Leydig cell recovery (n=3). Lines indicate mean and quartiles. **(E)** Schematic representation of transplantation of $Tcf21^{lin}$ cells following chemical ablation of Leydig cells in C57BL/6 animals. **(F)** Representative images of EDS treated C57BL/6 testes collected 24 hours and 7 days after transplant of $SCA1^{+}/cKIT^{-}$ cells from *Tcf21^{mCrem}:R26R^{tdTom}* animals, co-immunostained with SF1/TdTom (24 hrs n=4. 7 days n=6 per condition). Scale bars: 20 μm for each column. All images within a column were taken with the same magnification.

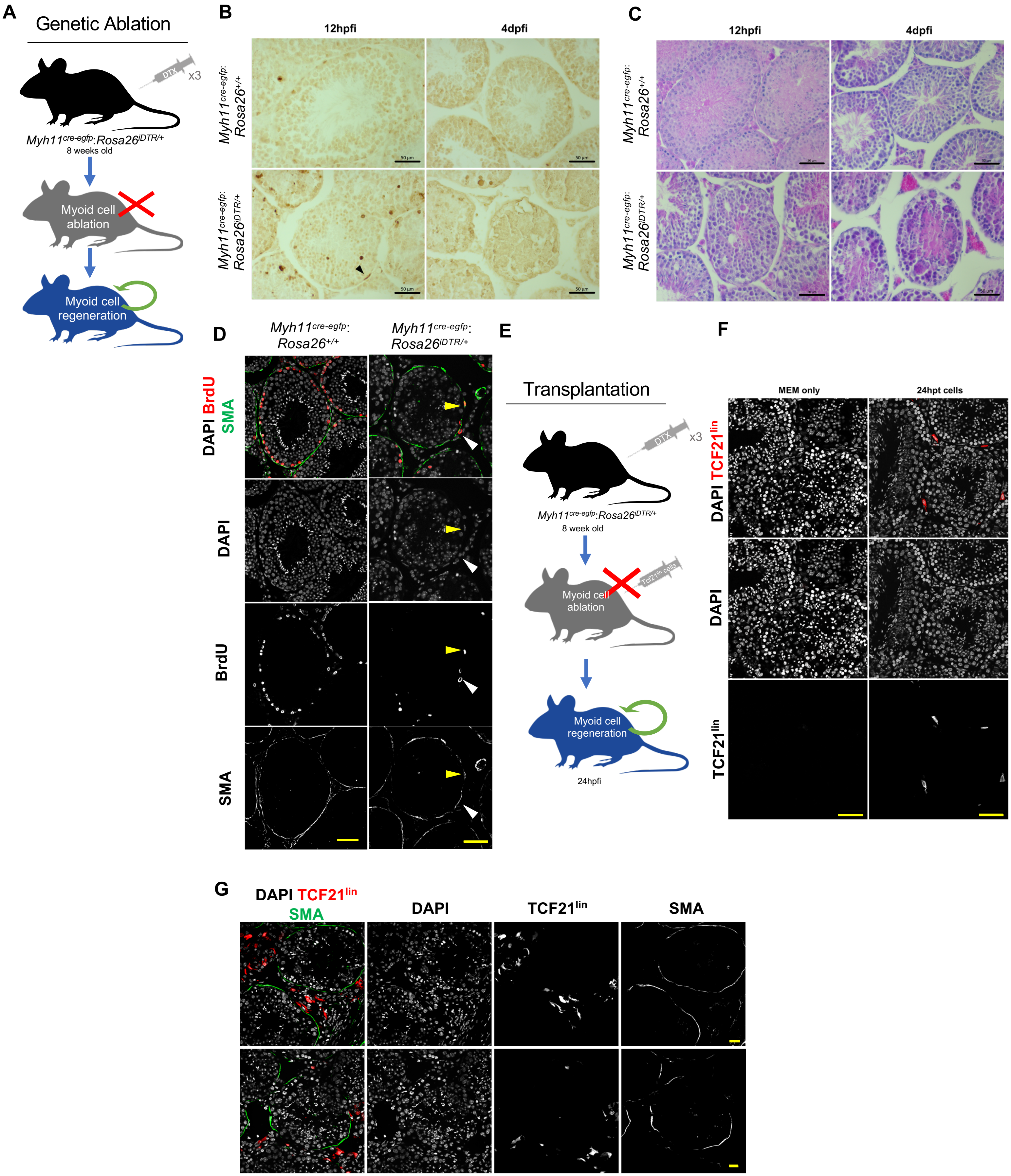
A
Genetic Ablation
DTX
x3
Myh11cre-egfp:Rosa26iDTR/+
8 weeks old
Myoid cell ablation
Myoid cell regeneration
B
12hpfi
4dpfi
Myh11cre-egfp: Rosa26+/+
Myh11cre-egfp: Rosa26iDTR/+
50 µm
C
12hpfi
4dpfi
Myh11cre-egfp: Rosa26+/+
Myh11cre-egfp: Rosa26iDTR/+
D
Myh11cre-egfp: Rosa26+/+
Myh11cre-egfp: Rosa26iDTR/+
DAPI BrdU SMA
DAPI
BrdU
SMA
E
Transplantation
DTX
x3
Myh11cre-egfp:Rosa26iDTR/+
8 week old
Tcf21lin cells
Myoid cell ablation
Myoid cell regeneration
24hpfi
F
MEM only
24hpt cells
DAPI TCF21lin
DAPI
TCF21lin
G
DAPI TCF21lin SMA
DAPI
TCF21lin
SMA

Supplementary Figure 6: Peritubular myoid cells can regenerate after Diphtheria toxin treatment and transplanted TCF21^{lin}cells home to the seminiferous tubule basement membrane after injury.

(A) Schematic representation of the genetic method used to ablate myoid cells. **(B-E)** Efficacy of diphtheria toxin treatment on *Myh11$^{cre\text{-}egfp}$*:*Rosa26$^{iDTR/+}$*or *Myh11$^{cre\text{-}egfp}$*:*Rosa26$^{+/+}$* animals. **(B)** Representative images of apoptotic myoid cell death by TUNEL staining in testes collected 12hpfi (n=4 per genotype) or 4dpfi (n=2 per genotype) of DTX. Scale bar: 50 μm. **(C)** Representative H&E staining at 12hpfi (n=3) or 4dpfi (n=2) of DTX. Scale bar: 100 μm**. (D)** Representative images of BrdU^{+}/SMA^{+} (yellow arrows) or BrdU^{+}/SMA^{-} (white arrows)at 4dpfi in *Myh11$^{cre\text{-}egfp}$*:*Rosa26$^{+/+}$*(n=3) or *Myh11$^{cre\text{-}egfp}$*:*Rosa26$^{iDTR/+}$*(n=2) animals. Scale bar: 20 μm**.** **(E)** Schematic representation of the TCF21^{lin}cell transplant following genetic ablation of myoid cells in *Myh11$^{cre\text{-}egfp}$*:*Rosa26$^{iDTR/+}$*animals. **(F)** Representative immunofluorescence images of TCF21^{lin} in *Myh11$^{cre\text{-}egfp}$*:*Rosa26$^{iDTR/+}$* animals at 24hpt (n=1 for MEM vehicle, n=3 for cells). **(D)** and **(F)** Scale bar: 20 μm for each column. All images within a column were taken at the same magnification. **(G)** Two representative immunofluorescence images of TCF21^{lin} (red) and SMA (green) in *Myh11$^{cre\text{-}egfp}$*:*Rosa26$^{iDTR/+}$* animals at 24hpt (n=3). Scale bar: 20 μm for each row. All images within a row were taken with the same magnification.

Supplementary Table 1. A summary of the conversion efficiency of various $TCF21^{lin}$ subtypes to either myoid or Leydig cells

Markers sorted	No. colonies used for Leydig differentiation	No. converted to Leydig	% Conversion	No. colonies used for Myoid differentiation	No. converted to myoid	% Conversion
$TCF21^{lin}/cKIT^{-}$	19	19	100%	13	13	100%
$TCF21^{lin}/SCA1^{+}/cKIT^{-}$	17	17	100%	6	6	100%
$TCF21^{lin}/SCA1^{+}/CD105^{-}/cKIT^{-}$	22	22	100%	14	14	100%
$TCF21^{lin}/SCA1^{+}/CD105^{+}/cKIT^{-}$	12	12	100%	6	6	100%
