# Supplementary material for: TCF21+ mesenchymal cells contribute to testis somatic cell development, homeostasis, and regeneration in mice
Source: Nat Commun. 2021 Jun 23;12:3876. doi: 10.1038/s41467-021-24130-8 (PMC8222243; doi:10.1038/s41467-021-24130-8)
Supplement: Supplementary file 2 — Description of Additional Supplementary Files [file 41467_2021_24130_MOESM2_ESM.pdf]

Description of Additional Supplementary Files

Supplementary Data 1. Markers for Somatic Cell Types (Related to Figure 1)

A. Markers for 6 Somatic Cell Types (Related to Figure 1A and 1B). A 1-sided binomial test was applied and adjustments were made for multiple comparisons.

B. Markers for 4 Somatic Cell Types (Related to Figure 1C and 1D). A 1-sided binomial test was applied and adjustments were made for multiple comparisons.

C. GO Term Enrichment for TCF21+ Int Population (Related to Figure 1D). A Fisher's exact test was applied with false discovery rate correction.

Supplementary Data 2. Markers for In-vitro Leydig Differentiation (Related to Figure 3)

A. Markers for 7 clusters of in-vitro Leydig differentiation. A 1-sided binomial test was applied and adjustments were made for multiple comparisons.

B. Gene expression centroids of 7 clusters of in-vitro Leydig differentiation.

Supplementary Data 3. Markers for Somatic Cell Types (Related to Figure 6)

A. Interaction scores between *Tcf21*$^{+}$ Interstitial cells and all other somatic cells in adult mouse testis, calculated by multiplying the centroid values for each cell type from scRNAseq dataset from Green et al. Top 5% of scores are shown, others are truncated to 0.

B. Interaction scores between *Tcf21*$^{+}$interstitial cells and spermatogonia, calculated in the same way as A.

Supplementary Data 4. Primers used for mouse genotyping

A. All primers used for mouse genotyping in this study

Supplementary Data 5. List of primary antibodies and concentrations used.
