# Supplementary material for: TCF21+ mesenchymal cells contribute to testis somatic cell development, homeostasis, and regeneration in mice
Source: Nat Commun. 2021 Jun 23;12:3876. doi: 10.1038/s41467-021-24130-8 (PMC8222243; doi:10.1038/s41467-021-24130-8)
Supplement: Supplementary file 8 — Reporting Summary [file 41467_2021_24130_MOESM8_ESM.pdf]

nature research

Corresponding author(s): Sue Hammoud

Last updated by author(s): May 23, 2021

Reporting Summary

Nature Research wishes to improve the reproducibility of the work that we publish. This form provides structure for consistency and transparency in reporting. For further information on Nature Research policies, see our Editorial Policies and the Editorial Policy Checklist.

Statistics

For all statistical analyses, confirm that the following items are present in the figure legend, table legend, main text, or Methods section.

n/a	Confirmed	
☐	☒	The exact sample size (*n*) for each experimental group/condition, given as a discrete number and unit of measurement
☐	☒	A statement on whether measurements were taken from distinct samples or whether the same sample was measured repeatedly
☐	☒	The statistical test(s) used AND whether they are one- or two-sided<br>*Only common tests should be described solely by name; describe more complex techniques in the Methods section.*
☒	☐	A description of all covariates tested
☐	☒	A description of any assumptions or corrections, such as tests of normality and adjustment for multiple comparisons
☐	☒	A full description of the statistical parameters including central tendency (e.g. means) or other basic estimates (e.g. regression coefficient) AND variation (e.g. standard deviation) or associated estimates of uncertainty (e.g. confidence intervals)
☐	☒	For null hypothesis testing, the test statistic (e.g. *F*, *t*, *r*) with confidence intervals, effect sizes, degrees of freedom and *P* value noted<br>*Give P values as exact values whenever suitable.*
☒	☐	For Bayesian analysis, information on the choice of priors and Markov chain Monte Carlo settings
☒	☐	For hierarchical and complex designs, identification of the appropriate level for tests and full reporting of outcomes
☐	☒	Estimates of effect sizes (e.g. Cohen's *d*, Pearson's *r*), indicating how they were calculated

Our web collection on statistics for biologists contains articles on many of the points above.

Software and code

Policy information about availability of computer code

Data collection	Images were collected using ZeissAX10 epifluorescence microscope, a Leica Leitz DMRD microscope, a Leica SP8 confocal microscope,or a Nikon A1R-HD25 confocal microscope as indicated in the Methods section.
Data analysis	Images were analyzed with ImageJ/ Fiji version 1.52g and GraphPad Prism version 8 was used for statistical analysis.<br>scRNAseq data were analyzed using Drop-seq_tools (v1.12), picard (v2.6.0), samtools (v1.2), STAR (v2.5.2b), R (v3.4.1), Seurat (v2.3.4), seriation (v1.2-2), and Monocle (v2.10.2) as described in the Methods.

For manuscripts utilizing custom algorithms or software that are central to the research but not yet described in published literature, software must be made available to editors and reviewers. We strongly encourage code deposition in a community repository (e.g. GitHub). See the Nature Research guidelines for submitting code & software for further information.

Data

Policy information about availability of data

All manuscripts must include a data availability statement. This statement should provide the following information, where applicable:

- Accession codes, unique identifiers, or web links for publicly available datasets
- A list of figures that have associated raw data
- A description of any restrictions on data availability

All relevant data are available from the corresponding authors. Raw and processed scRNAseq data files are available in GEO:GSE151337. Source data are provided with this paper for Figure 5E-G, 6C-D and Supplemental Figures S2B, S2I-J, S4C-H, S5D.

Field-specific reporting

Please select the one below that is the best fit for your research. If you are not sure, read the appropriate sections before making your selection.

[x] Life sciences   [ ] Behavioural & social sciences   [ ] Ecological, evolutionary & environmental sciences

For a reference copy of the document with all sections, see nature.com/documents/nr-reporting-summary-flat.pdf

Life sciences study design

All studies must disclose on these points even when the disclosure is negative.

Sample size	Effect size was not predetermined. Sample size was determined according to published literature investigating similar topics (one example being a study by Liu, et al. investigating the theca cell lineage in the ovary - https://doi.org/10.1038/ncomms7934 and another example being a study by Yokonishi, et al. investigating Sertoli cell ablation and regeneration - https://doi.org/10.1038/s41467-019-13879-8), to detect differences that are statistically significant. Statical significance was determined using T-test where needed.
Data exclusions	Poor quality cells from Single-cell RNA-seq data are filtered if they have less then 500 genes and >10% mitochondrial transcript. See Methods for greater detail.
Replication	All experiments were reproduced at least three times with independent biological samples unless otherwise specified in figure.
Randomization	For samples used in transplantation, gonads were randomly chosen for vehicle or experimental treatment (one gonad per condition). Otherwise, samples were grouped by genotype or treatment condition.
Blinding	Blinding was not possible.

Reporting for specific materials, systems and methods

We require information from authors about some types of materials, experimental systems and methods used in many studies. Here, indicate whether each material, system or method listed is relevant to your study. If you are not sure if a list item applies to your research, read the appropriate section before selecting a response.

Materials & experimental systems

n/a	Involved in the study	
[ ]	[x]	Antibodies
[x]	[ ]	Eukaryotic cell lines
[x]	[ ]	Palaeontology and archaeology
[ ]	[x]	Animals and other organisms
[x]	[ ]	Human research participants
[x]	[ ]	Clinical data
[x]	[ ]	Dual use research of concern

Methods

n/a	Involved in the study	
[x]	[ ]	ChIP-seq
[ ]	[x]	Flow cytometry
[x]	[ ]	MRI-based neuroimaging

Antibodies

Antibodies used

anti-Ly6a-Alexa Fluor 488 (Biolegend Cat#: 108115 ; RRID:AB_493270)
anti-CD73-APC (Biolegend Cat#: 127209 ; RRID:AB_11219400)
anti-CD90.1-Brilliant Violet 650 (Biolegend Cat#: 202533 ; RRID:AB_2562254)
anti-CD29-PE/Dazzle 594 (Biolegend Cat#:102231 ; RRID:AB_2716169)
anti-CD105-PerCP/Cy5.5 (BiolegendCat#:120415 ; RRID:AB_2562991)
anti-CD45-Brilliant Violet 510 (Biolegend Cat#: 103137 ; RRID:AB_2561392)
anti-CD117-PE/Cy7 (c-KIT) (Biolegend Cat#: 105813 ; RRID: AB_313222)
anti-CD34-PE (Biolegend Cat#:128609 ; RRID: AB_207460)
Anti- FGF5 (Santa Cruz Cat#:sc-376264 ; RRID: AB_10985985)
Anti-SMA (Sigma Cat#:A5228 ; RRID:AB_262054)
Anti-CD34 (Abcam Cat#:ab81289 ; RRID:AB_1640331)
Anti-Cyp17A1 (Abcam Cat#:ab80206, RRID:AB_1603486)
Anti-Cyp17A1 (ProteinTech Cat#:14447-1-AP ; RRID: AB_2292527)
Anti-Cyp17A1 (Santa Cruz Cat#:sc-374244 ; RRID:AB_10988393)
Anti-CoupTFII (R&D Systems Cat#:PPH7147-00 ; RRID:AB_567523)
Anti-Foxl2 (A gift from Dagmar Wilhelm, University of Melbourne, Australia ; Anti-FOXL2_rabbit A ; RRID:AB_2687958)
Anti-Foxl2 (Novus Biological Cat#:NB100-1277 ; RRID:AB_2106188)
Anti-dsRed (tdTomato) (Takara Bio USA Cat#:632496; RRID:AB_10013483)

	Anti-3βHSD (Santa Cruz Cat#:sc-30820 ; RRID:AB_2279878)<br>Anti-WT1 (Santa Cruz Cat#:sc-192 ; RRID:AB_632611)<br>Anti-PECAM (CD31) (BD Biosciences Cat#:553370 ; RRID:AB_394816)<br>Anti-SF1 (Cosmo Bio Cat#:KAL-KO610)<br>Anti-SF1 (A gift from Gary Hammer, University of Michigan ; Cat#:CustomSf1 ;RRID: AB_2716716)<br>Anti-SOX9 (EMD-Millipore, Cat#:ABE571)<br>Anti-SOX9 (Abcam, Cat#:ab3697, RRID:AB_304012)<br>Anti-VASA (DDX4) (Abcam Cat#:ab13840, RRID:AB_443012)<br>Anti-GFP (Abcam Cat#:ab6556 ; RRID:AB_305564)<br>Anti-B-actin (Novus Cat#:NBP1-47423 ; RRID:AB_10010376)<br>Anti-BrDU (Abcam Cat#:ab6326 ; RRID:AB_2313786)<br>Anti-GAPDH (ProteinTech Cat#:10494-1-AP ; RRID:AB_2263076)<br>Anti-Osterix (Abcam Cat#ab22552 ; RRID:AB_2194492)<br>Anti-Perilipin (Sigma Cat# P1873 ; RRID:AB_532267)
Validation	Commercially available antibodies have provided validation statements on their respective websites.<br>CustomSf1: Sonic Hedgehog and WNT Signaling Promote Adrenal Gland Regeneration in Male Mice. Finco et al, Endocrinology 2018.<br>Anti-FOXL2_rabbit A: Sox10 gain-of-function causes XX sex reversal in mice: implications for human 22q-linked disorders of sex development. Polanco et al, Human Molecular Genetics 2010.<br>Testis Determination Requires a Specific FGFR2 Isoform to Repress FOXL2, Bagheri-Fam et al. Endocrinology 2017.

Animals and other organisms

Policy information about studies involving animals; ARRIVE guidelines recommended for reporting animal research

Laboratory animals	Various mouse (Mus musculus) strains, transgenic, and/or mixed genetic backgrounds listed below were used to obtain testis (male) or ovary (female) samples between stages embryonic (E) 10.5-E17.5 for fetal studies, 5 weeks for juvenile, and 6-18 weeks for adult studies. Details can be found in manuscript and methods.<br><br>C57BL/6 (The Jackson Laboratory #000664)<br>Rosa-LSL-tdTomato (B6.Cg-Gt(ROSA)26Sortm9(CAG-tdTomato)Hze/J (The Jackson Laboratory #007909)<br>Oct4-eGFP (B6;CBA-Tg(Pou5f1-EGFP)2Mnn/J) (The Jackson Laboratory #004654)<br>Gli1-egfp (Gurdziel et al. (2016))<br>Prgfra-egfp (B6.129S4-Pdgfratm11(EGFP)Sor/J) (The Jackson Laboratory #007669)<br>Tcf21-icre (Acharya et al. (2011))<br>CD1 (CD-1® IGS) (Charles River Laboratories #022)<br>MYH11cre-Egfp (The Jackson Laboratory #007742)<br>Rosa26iDTR (The Jackson Laboratory #007900)
Wild animals	Our study did not involve wild animals.
Field-collected samples	Our study did not involve field-collected samples.
Ethics oversight	All animal experiments were carried out with prior approval of the University of Michigan Institutional Committee on Use and Care of Animals (Animal Protocols: PRO00006047, PRO00008135), in accordance with the guidelines established by the National Research Council Guide for the Care and Use of Laboratory Animals.

Note that full information on the approval of the study protocol must also be provided in the manuscript.

Flow Cytometry

Plots

Confirm that:

- [x] The axis labels state the marker and fluorochrome used (e.g. CD4-FITC).
- [x] The axis scales are clearly visible. Include numbers along axes only for bottom left plot of group (a 'group' is an analysis of identical markers).
- [x] All plots are contour plots with outliers or pseudocolor plots.
- [x] A numerical value for number of cells or percentage (with statistics) is provided.

Methodology

Sample preparation	Sample preparation described in manuscript and methods.
Instrument	Sony Synergy
Software	FlowJo

Cell population abundance	Single cell RNAseq identified a small number of endothelial cells ~6%.<br>Abundance of Sca1 and Tcf21-lin cells within each population shown in Figure S2G.
Gating strategy	Gating strategy used to characterize marker expression in interstitial Sca1+ or Tcf21lin cells in the testis employs side scatter area and CD45 fluorescent intensity used to eliminate immune cell populations (selection of Sca1+ or Tcf21lin+, CD45-), followed by selection of cKit- cells to exclude mature Leydig cells. Positive gates were determined using FMO isotype controls. Isotype controls were used for additional markers.

☒ Tick this box to confirm that a figure exemplifying the gating strategy is provided in the Supplementary Information.
