# Supplementary material for: TCF21+ mesenchymal cells contribute to testis somatic cell development, homeostasis, and regeneration in mice
Source: Nat Commun. 2021 Jun 23;12:3876. doi: 10.1038/s41467-021-24130-8 (PMC8222243; doi:10.1038/s41467-021-24130-8)
Supplement: Supplementary file 9 — Source Data [file 41467_2021_24130_MOESM9_ESM.zip › Source Data/Figure S5_western blot.pdf]

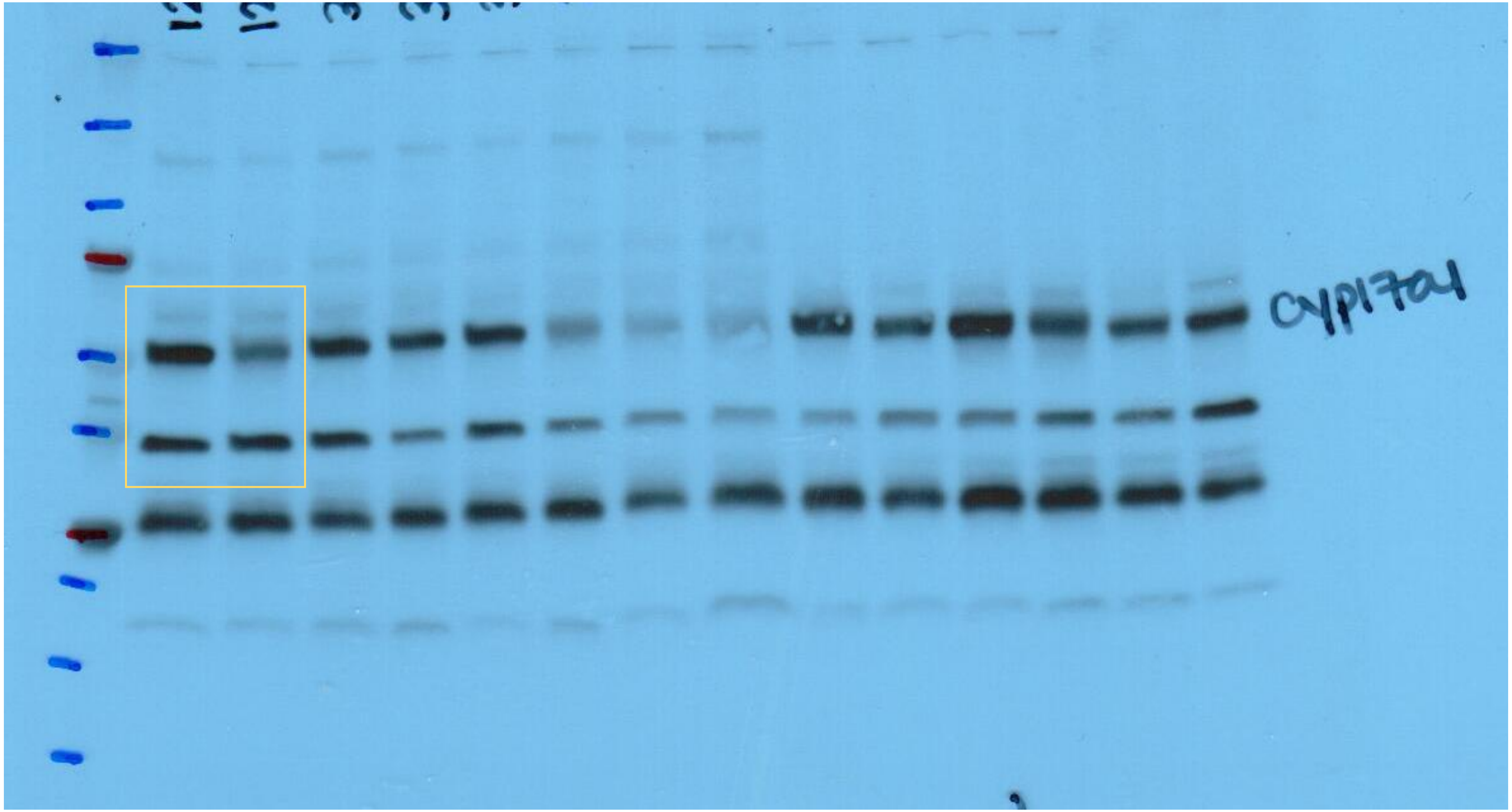

Figure S5
Used for 24hr western

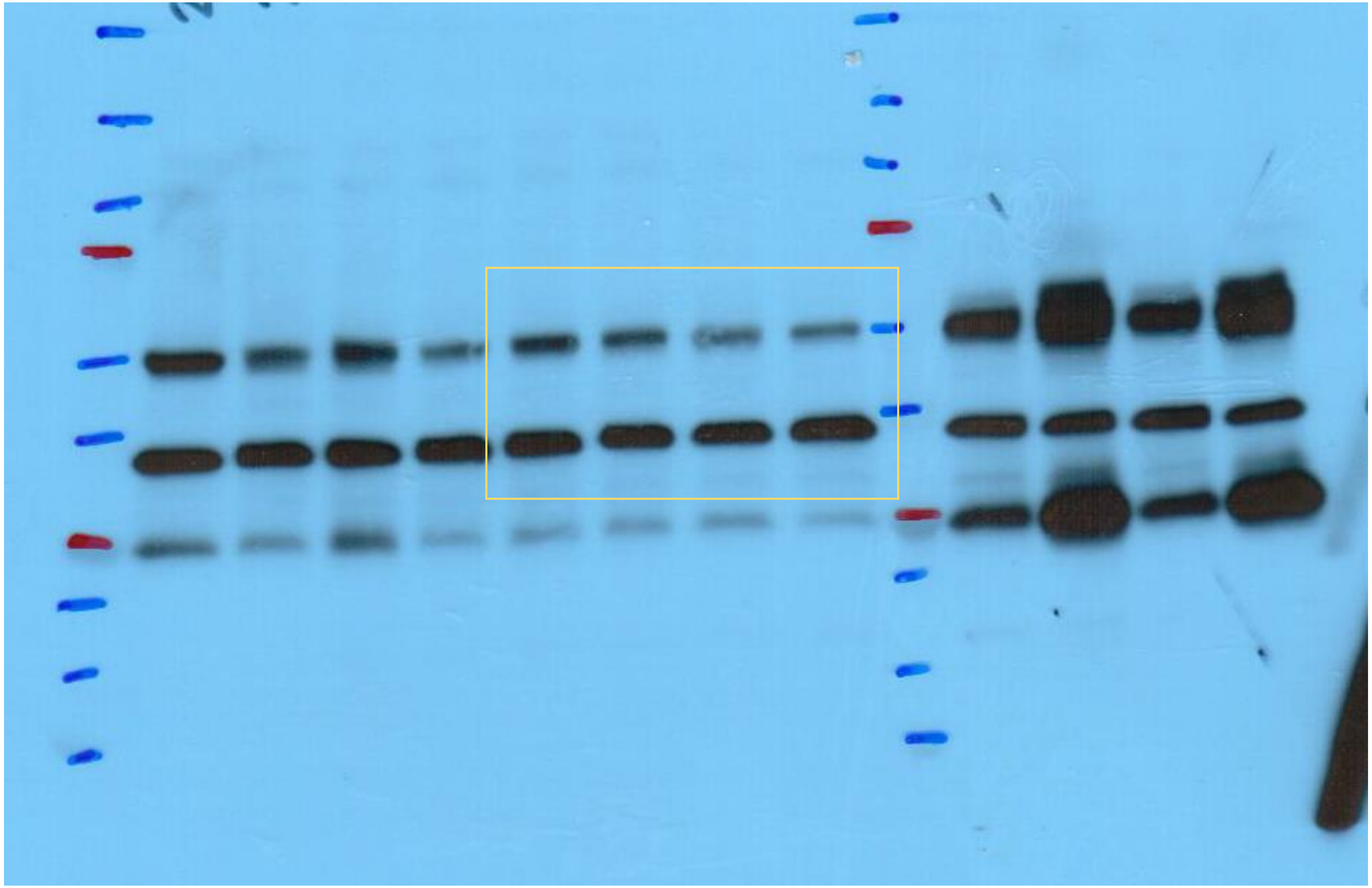

Figure S5
Used for 3d western

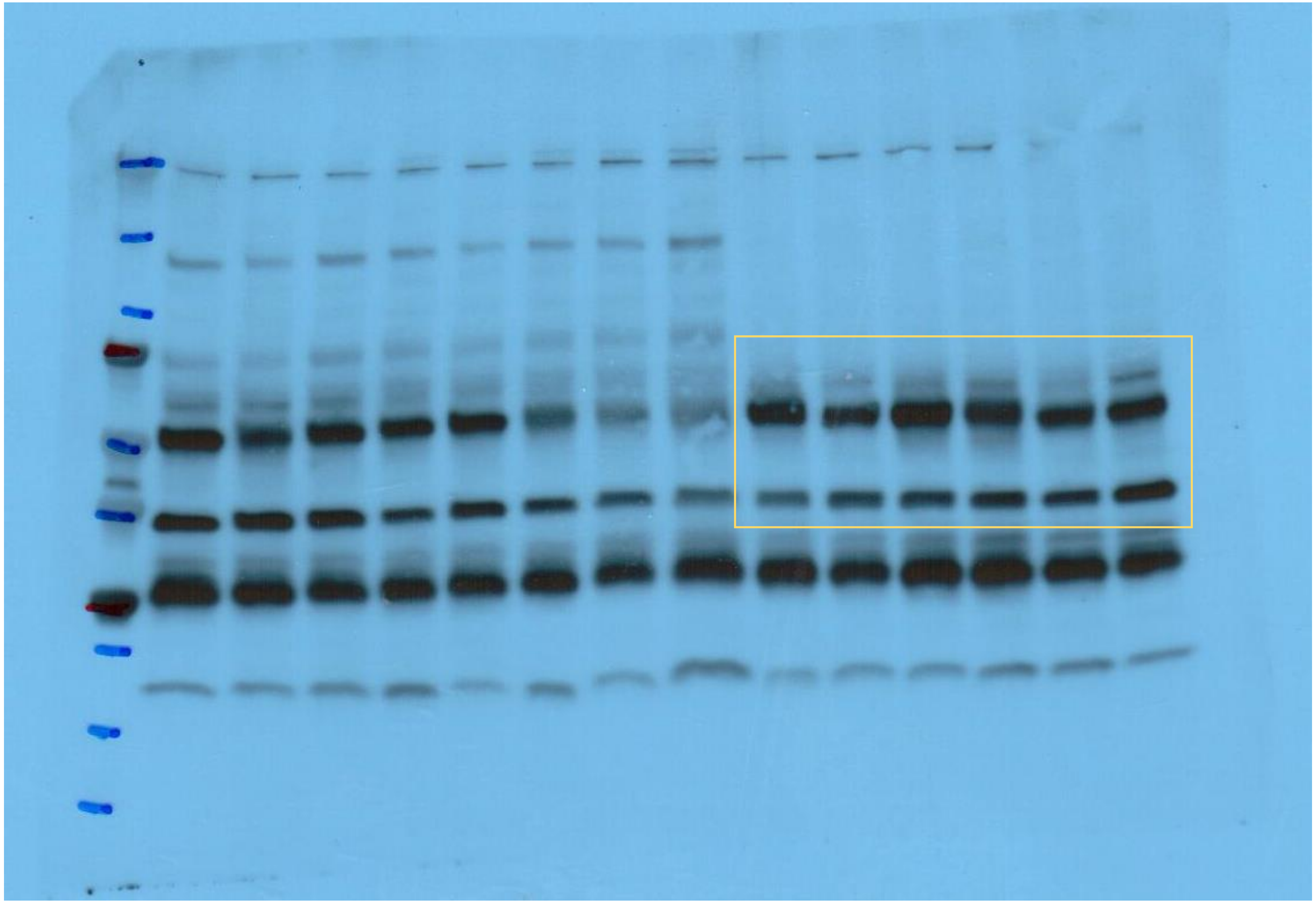

Figure S5
Used for 7d western

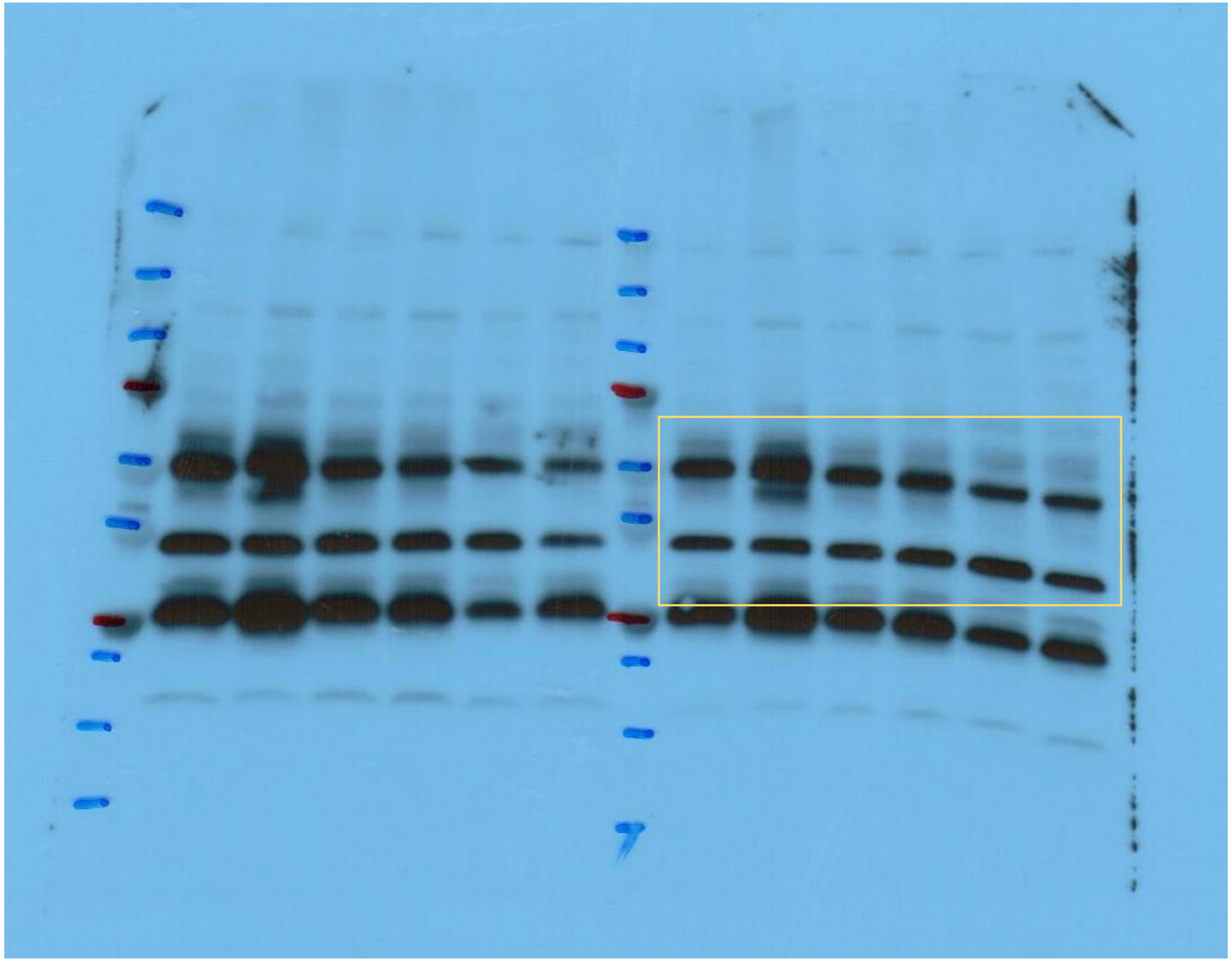

Figure S5
Used for 14d western
