# Supplementary figures and images for: TCF21+ mesenchymal cells contribute to testis somatic cell development, homeostasis, and regeneration in mice
Source: Nat Commun. 2021 Jun 23;12:3876. doi: 10.1038/s41467-021-24130-8 (PMC8222243; doi:10.1038/s41467-021-24130-8)

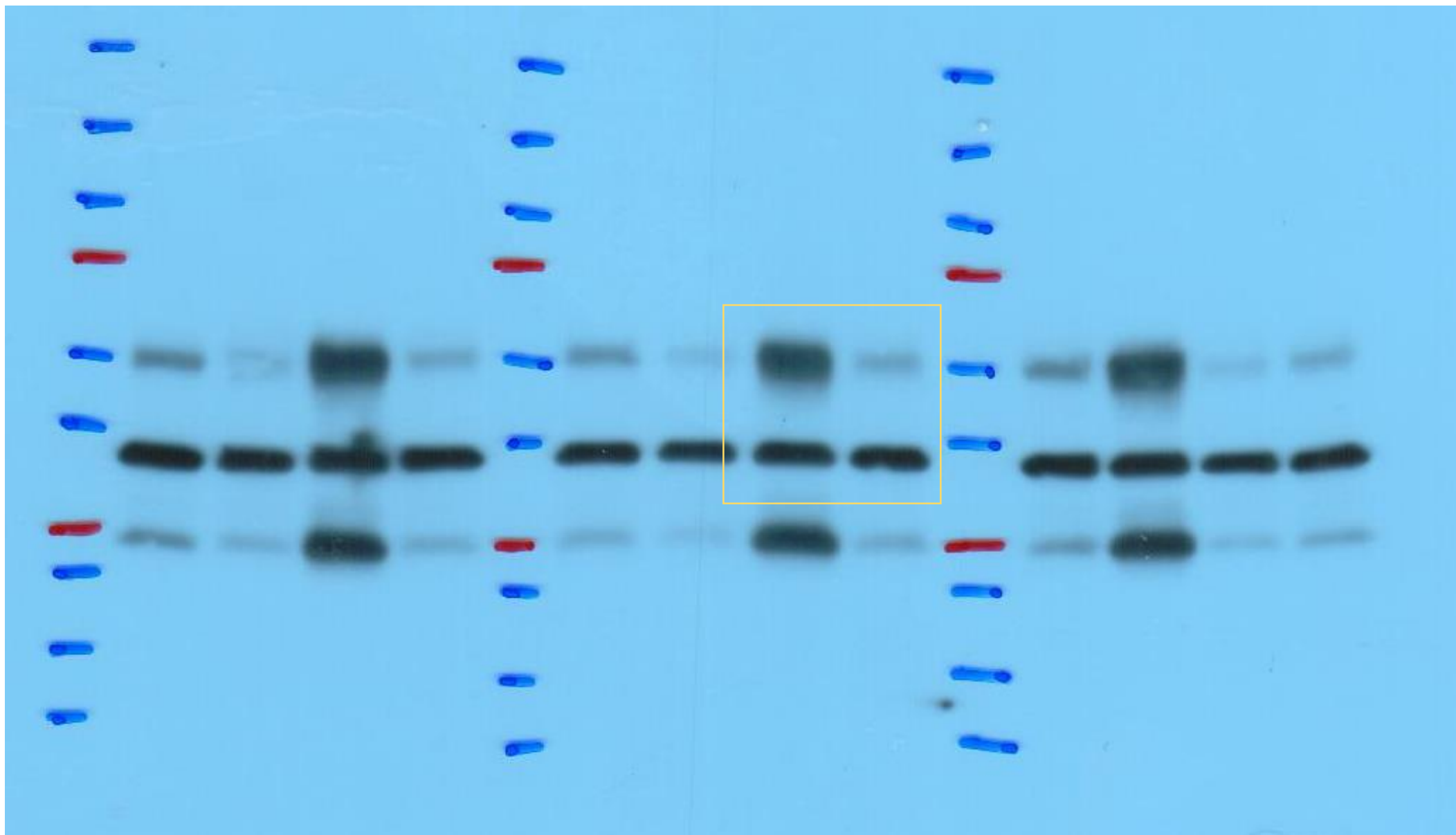

Fig 5  
Used for 3dpfi

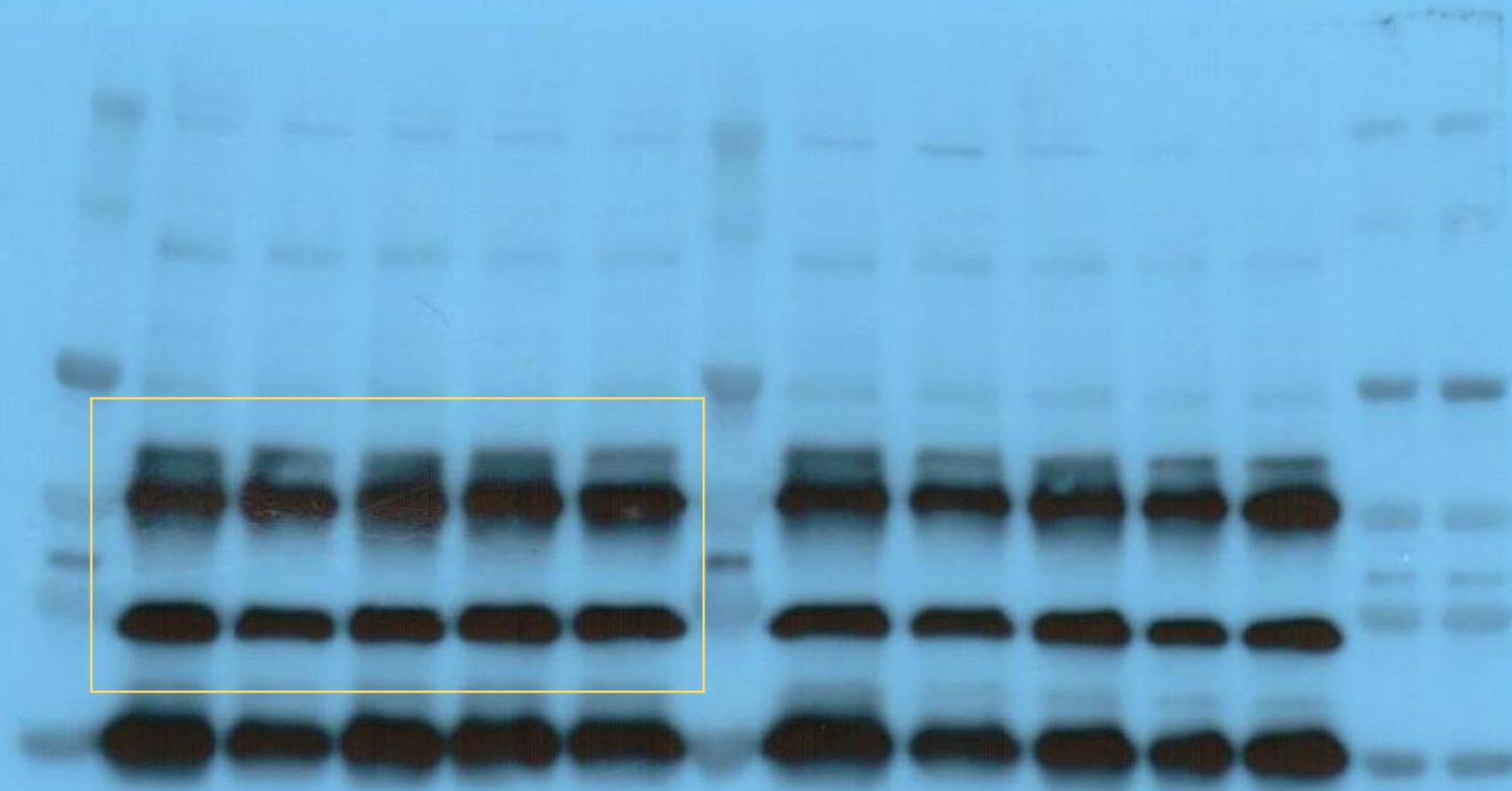

Fig 5  
Used for 24dpi

Supplement: Supplementary file 9 — Source Data [file 41467_2021_24130_MOESM9_ESM.zip › Source Data/Figure 5_western blot.pdf]
